# High-resolution structure of monomeric photosystem I from *Thermosynechococcus vestitus*

**DOI:** 10.1007/s11120-026-01237-x

**Published:** 2026-10-15

**Authors:** Jelena Boyka, Guillaume Gaullier, Jack Forsman, André T. Graça, Maksym Golub, Nicholas Croy, Kathleen Wood, Anthony P. Duff, Michael Moir, Jörg Pieper, Johannes Messinger, Frank Müh, Athina Zouni, Cecilia Blikstad

**Affiliations:** 1 Department of Biology, Humboldt-Universität zu Berlin, 10099 Berlin, Germany; 2 Department of Chemistry - Ångström Laboratory, Uppsala University, Uppsala, 75120 Sweden; 3 Umeå Plant Science Centre, Department of Plant Physiology, Umeå University, Umeå, 90817 Sweden; 4 Institute of Physics, University of Tartu, Tartu, 50411 Estonia; 5 Australian Centre for Neutron Scattering, Australian Nuclear Science and Technology Organization, Lucas Heights, NSW 2234 Australia; 6 National Deuteration Facility, Australian Nuclear Science and Technology Organization, Lucas Heights, NSW 2234 Australia; 7 Department of Theoretical Biophysics, Institute for Theoretical Physics, Johannes Kepler University Linz, Linz, 4040 Austria; 8 Present Address: Department of Biochemistry, University of Otago, Dunedin, 9016 New Zealand; 9 Present Address: European Molecular Biology Laboratory (EMBL), EMBL Grenoble, Grenoble, 38042 France

**Keywords:** Photosynthesis, Photosystem I, Cytochrome c_6_, Structure, Cryo-EM, Small angle neutron scattering

## Abstract

Photosystem I (PSI) exists in trimeric (tPSI) and monomeric (mPSI) forms within the thylakoid membrane of cyanobacteria. PSI functions as a light-driven electron pump, enabling the transfer of electrons from cytochrome *c*_6_ (Cyt *c*_6_) or plastocyanin to ferredoxin. Here, we have developed a novel protocol to obtain large amounts of intact, active mPSI from *Thermosynechococcus vestitus* (*Tv*) by dissociating highly purified tPSI using the detergent octaethylene glycol monododecyl ether (C_12_E_8_) at elevated temperatures. We analysed the stability of the protein-detergent complex employing a combination of spectroscopic methods, such as fluorescence at 77 K and circular dichroism (CD), and investigated the detergent’s influence on delipidation and monomerisation. We determined the structure of mPSI to a resolution of 1.8 Å using cryogenic electron microscopy (cryo-EM). This structure retains all subunits except PsaL and we identified a sulfoquinovosyl diacylglycerol (SQD) molecule and 16 n-dodecyl ß-maltoside (βDM) molecules arranged in a ring around the complex. The mPSI structure was verified in solution at physiological temperature by applying small angle neutron scattering. Comparing the cofactor inventory of mPSI to that of tPSI, we suggest the assignment of a red-shifted chlorophyll pigment and discuss the influence of detergents and lipids on stability and oligomeric state of PSI. Finally, when adding native *Tv* Cyt *c*_6_ under reducing conditions, additional density was observed in close proximity of the P_700_ pigments, which indicates the presence of a transiently bound Cyt *c*_6_.

**Supplementary Information:**

The online version contains supplementary material available at https://doi.org/10.1007/s11120-026-01237-x .

## Introduction

The processes of photosynthesis are imperative for the generation of vital oxygen in the Earth’s atmosphere. The conversion of solar energy into stored chemical energy in the form of carbohydrates in cyanobacteria, algae, and higher plants is achieved through the activity of two membrane proteins: photosystem I (PSI) and II (PSII) (Barber 2004; Nelson 2011). PSI effectuates the light-driven transfer of an electron from reduced plastocyanin (Pc) or cytochrome (Cyt) *c*_6_ to oxidised ferredoxin (Fd) (Golbeck 2006; Nelson and Yocum 2006). More particularly, this electron transfer from the native electron donor of *Thermosynechococcus vestitus*, Cyt *c*_6_, is initiated under reducing conditions, and Cyt *c*_6_ is only transiently binding to PSI (Kölsch et al. 2020). Nevertheless, its binding is believed to inflict conformational changes to the trimeric PSI (tPSI) (Li et al. 2022). The role of Cyt *c*_6_ is to deliver electrons to P_700_, a special pair of chlorophyll (Chl) co-factors (Chl_A1/B1_) in the reaction centre (RC) of PSI, from which the light-induced charge separation starts, resulting in an electron entering one of the two pseudo-symmetric branches (annotated as “A” or “B”; A-branch preferred (Dashdorj et al. 2005)) of the electron transfer chain (ETC). The ETC leads from P_700_ over two successive chlorophylls (Chl_A2/B2_ and Chl_A3/B3_) to a phylloquinone (PQN) on each branch and, finally, to the first of three iron sulphur clusters (FeS), F_x_ (Fromme et al. 2001; Jordan et al. 2001; Şener et al. 2011). Afterwards, the FeS F_A_ and then F_B_ are reduced before the electron is transferred to Fd. Electrons from reduced Fd are directly utilised to reduce nutrients or to produce the more stable biological reductant NADPH.

Previously, dynamic femtosecond (fs) X-ray free electron laser (XFEL) crystallography measurements on tPSI crystals provided functional insights into light-driven electron transfer (ET) in the cyanobacterium *Thermosynechococcus vestitus* (formely *T. elongatus*) PSI under physiological conditions (Fromme 2015; Keable et al. 2021). However, the mechanisms underlying charge separation and the dynamic ET reaction in the PSI-RC remain to be fully elucidated (Fromme et al. 2006; Fromme and Spence 2011; Fromme 2015; Gisriel et al. 2019; Keable et al. 2021).

In the thylakoid membrane, PSI exists in trimeric and monomeric forms. The majority of PSI X-ray structures are based on type II crystals containing detergent belts consisting of n-dodecyl ß-maltoside (βDM) around the protein complexes (Jordan et al. 2001; Ben-Shem et al. 2004; Mazor et al. 2014, 2015). βDM is a mild, non-ionic detergent and, consequently, the most commonly used detergent for extracting PSI from the native membrane and keeping it stably solubilised as a protein-detergent complex (PDC) in an aqueous environment (Loll et al. 2005; Umena et al. 2011; Parker and Newstead 2012; Mazor et al. 2014, 2017; Golub et al. 2020, 2021, 2022). The first X-ray crystal structure of the trimeric ßDM PSI (ßDM tPSI) from the cyanobacterium *T. vestitus* at cryogenic temperature, resolved at 2.5 Å resolution, showed a homo-trimer with a mass of over 1 MDa (Jordan et al. 2001). Each monomer is comprised of 12 protein subunits (PsaA-F, PsaI-M and PsaX) and is equipped with 127 cofactors. The PsaL subunits function as an interface between the PSI monomers and, thus, play a role in monomerisation (Chitnis, V. P. and Chitnis, P. R. 1993; Xu et al. 1995; Mazor et al. 2014; Kłodawska et al. 2015; Netzer-El et al. 2018). In addition, previous research suggests that the lipids located in the monomer-monomer interface are also crucial in this respect (Malavath et al. 2018). The lipids in tPSI consist mainly of the neutral galactolipids monogalactosyl diacylglycerol (MGDG; ~45%) and digalactosyl diacylglycerol (DGDG; ~25%), and the anionic lipids sulphoquinovosyl diacylglycerol (SQDG; ~15–25%) and phosphatidyl glycerol (PG; ~5–15%) (Sakurai et al. 2006; Kern and Guskov 2011). Especially, the galactolipids (MGDG and DGDG) are believed to be exchanged to ßDM due to their structural similarity to the detergent (Guskov et al. 2009; Kern et al. 2009; Brady et al. 2022). However, the role of endogenous and peripheral lipids in and around the native PSI complex in the formation of its various oligomeric forms under different environmental conditions remains largely unclear (Guskov et al. 2009; Kern et al. 2009; Kern and Guskov 2011; Russo Krauss et al. 2013; Malavath et al. 2018; Brady et al. 2022; Golub and Boyka et al. 2025).

PSI can be used for numerous biotechnological processes due to its extremely high quantum efficiency of almost 100% and high stability, such as using the generated electrons for reduction (e.g., of CO_2_) or to produce, e.g., hydrogen H_2_ in photobioelectrodes consisting of enzymes coupled to PSI (Appel et al. 2020; Wang et al. 2023). Recently, Morlock et al. (2023) achieved successful light-driven CO₂ reduction to formate *via* the direct attachment of PSI coupled to formate dehydrogenase (FDH) to an electrode. Other photobiocathodes utilise bound non-native Cyt *c* from horse heart (Cyt *c*_HH_) to mediate more efficient electron transport (Stieger et al. 2016; Ciornii et al. 2017, 2019; Feifel et al. 2018; Morlock et al. 2021, 2022).

Further, monomeric PSI (mPSI) was tested to fill the gaps in a 3D-electrode covered with tPSI (Nioradze et al. 2021). In addition, the native Cyt *c*_6_-PSI complex has potential applications in biotechnology (Kargul et al. 2012). Although the binding affinity of Cyt *c*_6_ to PSI is weaker than the binding affinity of Cyt *c*_HH_ to the PSI complex, Cyt *c*_6_ binds to PSI at a conserved binding site and no other non-specific bindings occur (Kölsch et al. 2018). Nevertheless, the activity of native PSI-Cyt *c*_6_ is highest under physiological conditions (Kölsch et al. 2018).

The structures of complexes between PSI and its redox mediators, such as Fd-tPSI-Pc (Caspy et al. 2020), Fd-tPSI-Cyt *c*_6_ (Li et al. 2022) and cross-linked green algae (*Chlamydomonas reinhardtii)* mPSI:Cyt *c*_6_ (Ogawa et al. 2026), have been investigated using cryo-EM. However, the binding of native Cyt *c*_6_ to cyanobacterial (e.g. *T. vestitus*) PSI is yet to be fully elucidated (Kölsch et al. 2018). As postulated by Li et al. (2022) and Kölsch et al. (2018), the occupancy of Cyt *c*_6_ per monomer in tPSI might be less than one and, hence, too low to be detected in cryo-EM reconstructions (Kölsch et al. 2018; Li et al. 2022). Consequently, a PSI monomer instead of a trimer would facilitate 3D classification to separate mPSI-Cyt *c*_6_ complexes from free mPSI particles. Moreover, a unique feature of PSI – and a topic of high interest – is the presence of Chl *a* co-factors which absorb at energies lower than P_700_. These are referred to as low-energy Chls, long-wavelength Chls, or shortly “red Chls”. They are believed to be essential for light-energy absorption and its regulation (Skandary et al. 2015; Toporik et al. 2020; Çoruh et al. 2021). However, the role of the red chlorophylls in the excitation energy transfer (EET) in PSI remains poorly understood (Sohail et al. 2025). It is noteworthy that during procedures to form mPSI from cyanobacterial tPSI, red Chls are usually lost, which provides a means for a structural assignment of red Chls (Çoruh et al. 2021). Other methods employed in the literature to assign red Chls are the structural and spectroscopic comparison of various species with a different Chl inventory (Karapetyan et al. 2014; Malavath et al. 2018) and the genetic manipulation of the antenna protein to engineer Chl binding sites (Toporik et al. 2020).

Here, we present a novel protocol for producing large quantities of nearly intact and active *T. vestitus* mPSI from purified, active tPSI. Additionally, we present a cryo-EM structure of our mPSI at a resolution of 1.8 Å, and characterise mPSI compared to tPSI using absorption spectroscopy at room temperature (RT) and fluorescence spectroscopy at 77 K, providing information about the loss of carotenoids (ß-carotene) and red chlorophylls accompanying the monomerisation. We further investigate the oligomerisation mechanism and reveal a potential binding site of Cyt *c*_6_.

## Results

### Generation of functional monomeric PSI

Prior to our monomerisation protocol we used Blue Native (BN) PAGE (SI, Fig. S3), DLS (SI, Table S1), MALDI-ToF (SI, Fig. S4), and size exclusion chromatography (SI, Fig. S5), and confirmed that the pre-crystallised tPSI samples exhibit high homogeneity, monodispersity and the complete 12 subunit composition. Incubating ßDM tPSI for 30 min with the addition of 1% C_12_E_8_ at 60 °C yielded almost solely mPSI. The same result was achieved with C_12_E_8_ tPSI and further experiments showed that the incubation time could be shortened to 10 min and still similar results could be produced (SI, Fig. S1, S2). However, the PsaL subunit was absent in the rebuffered ßDM mPSI sample according to the MALDI-ToF spectra (SI, Fig. S4). In addition, the generated ßDM mPSI samples were analysed before structure determination by cryo-EM to ascertain homogeneity, monodispersity (Pd < 15%), size (SI, Table S1) and thermal stability (SI, Table S13). The functional integrity of our mPSI is demonstrated by the O_2_ activity rates, which are similarly high for mPSI and tPSI (SI, Table S1).

### ßDM mPSI cryo-EM structure

After employing our protocol for the monomerisation of tPSI, we solved a cryo-EM structure of *Tv* ßDM mPSI at 1.8 Å resolution. Note that the ßDM mPSI sample, which had C_12_E_8_ added during the monomerisation process, was transferred into a ßDM buffer (via sucrose gradient; SI, Fig. S2) before cryo-EM experiments. A total of 11 protein subunits could be identified in the structure, whereby only the subunit important for trimerisation, PsaL, is not detected, consistent with the mass spectrometry characterisation (SI, Fig. S4). Furthermore, the cryo-EM map revealed 1183 water molecules, 19 β-carotenes (ß-Car), 91 chlorophyll *a* (Chl *a*) molecules, seven lipids (five phosphatidyl glycerol (PG or LHG), one monogalactosyl diacylglycerol (MGDG or LMG), one sulfoquinovosyl diacylglycerol (SQDG or SQD) newly discovered in PSI), 16 ßDM detergent molecules (LMT), two phylloquinones (PQN), three iron-sulphur clusters (4Fe4S or SF4), and one Mg^2+^ ion. In our structure, we can clearly distinguish Chl *a* and the epimer Chl *a*’ in P_700_ (SI, Fig. S10c).

**Fig. 1:**
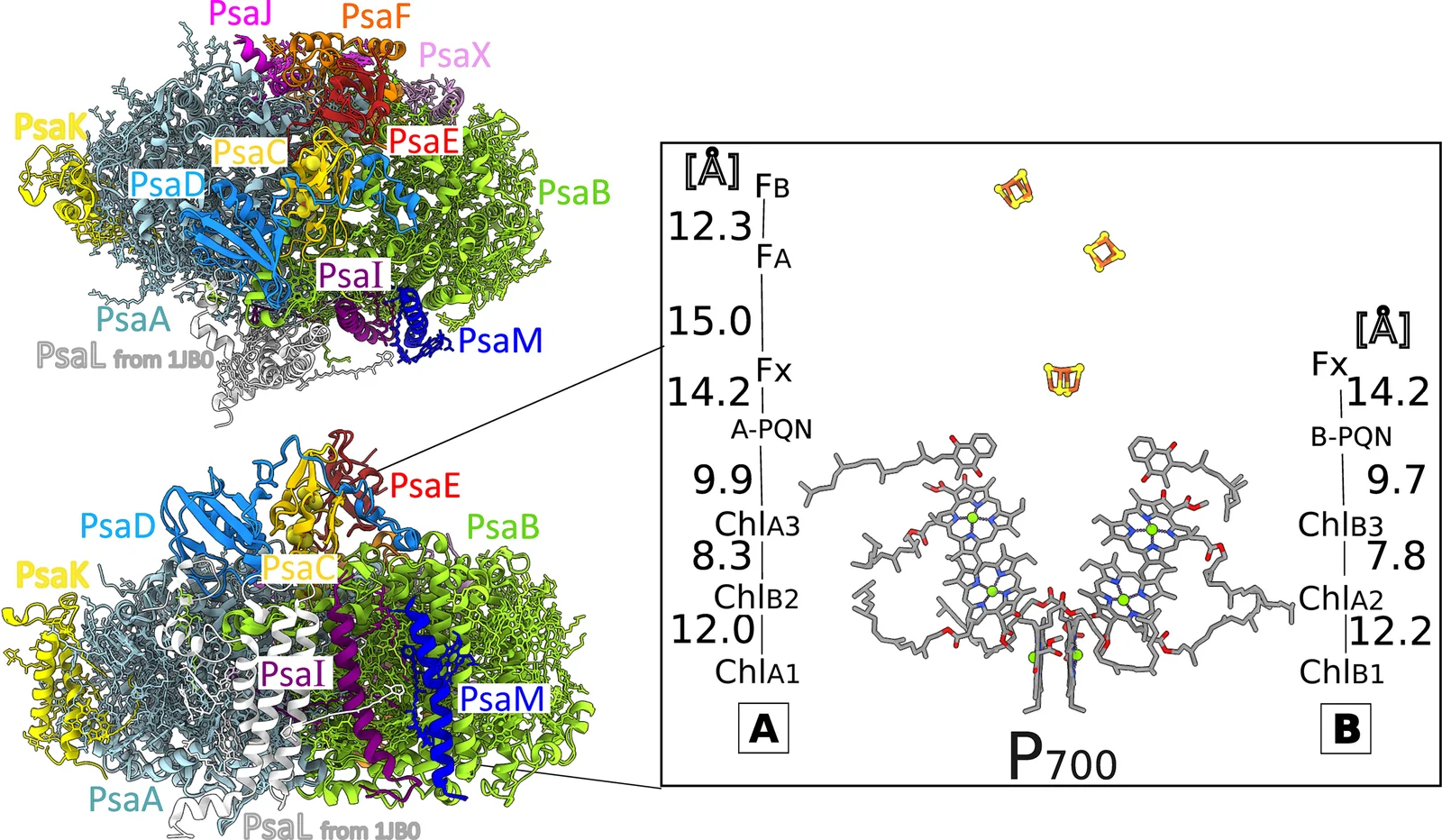
Overview of the cryo-EM structure of mPSI. *Upper left*: View on mPSI from the stromal side with 11 of the 12 subunits of PSI present. The subunit important for trimerisation, PsaL, is missing and shown here from 1JB0 (Jordan et al. 2001) in grey. *Lower left*: View on mPSI along the membrane plane with the stromal side up. Subunits as in the upper panel. *Right*: ETC with the two branches **A** and **B** and the centre-to-centre distances between the involved cofactors in Å. Distance measurement done as in Jordan et al. (2001), i.e. the centre of a Chl *a* is defined by the position of the central Mg^2+^-ion as well as between Mg atom and the centre of the phylloquinone carbonyls and the centre of the iron-sulphur clusters (PyMOL: distances to rings)

### Resolution, B-factor and ETC of our mPSI

The resolution at 1.8 Å is very homogeneous over the entire mPSI, but is slightly better in the cytoplasmic and lumenal regions compared to the transmembrane region. Most parts of mPSI seem to have a low flexibility as indicated by atomic B-factors (SI, Fig. S8), only PsaK and some cofactors or amino acid residues reaching into the membrane plane exhibit higher flexibility. The transmembrane regions of the mPSI are lipophilic and the luminal and cytoplasmic surfaces display mostly hydrophilic residues.

The electron transport chain (ETC) of PSI is composed of P_700_ (A1, B1), the Chl acceptors A2, B2 and A3, B3, the phylloquinones (PQN) as well as the three iron sulphur clusters F_X_, F_A_ and F_B_ (Fig. 1). The centre-to-centre distances of A1-B2 and B1-A2 are more similar in structures of the monomer than in those of a trimer (SI, Table S6 and Fig. S12), and all the ETC cofactors (except for P_700_) are coordinated/H-bonded by the same type of molecule symmetrically in each branch (SI, Tables S5, S6). In addition, the atomic B-factors (SI, Fig. S11) support this high similarity of both branches throughout the whole ETC of our mPSI structure. In fact, when comparing tPSI and mPSI structures in terms of the relative flexibility of both branches (SI, Fig. S11), the A-branch is more flexible than the B-branch in tPSI, whereas both branches are equally rigid in mPSI. All analysed structures other than our mPSI exhibit an increasing flexibility of the three iron sulphur clusters in the direction radially outwards of PSI, with F_B_ being the most flexible one. When overlaying both branches of our mPSI at the P_700_ Chls, we see A2 and B2 almost matching (0.3 Å), whereas the central Mg^2+^ ions in A3 and B3 are shifted by 0.8 Å and the rings of PQN by 1.4–1.7 Å (SI, Fig. S12).

### Carotenoids

**Fig. 2:**
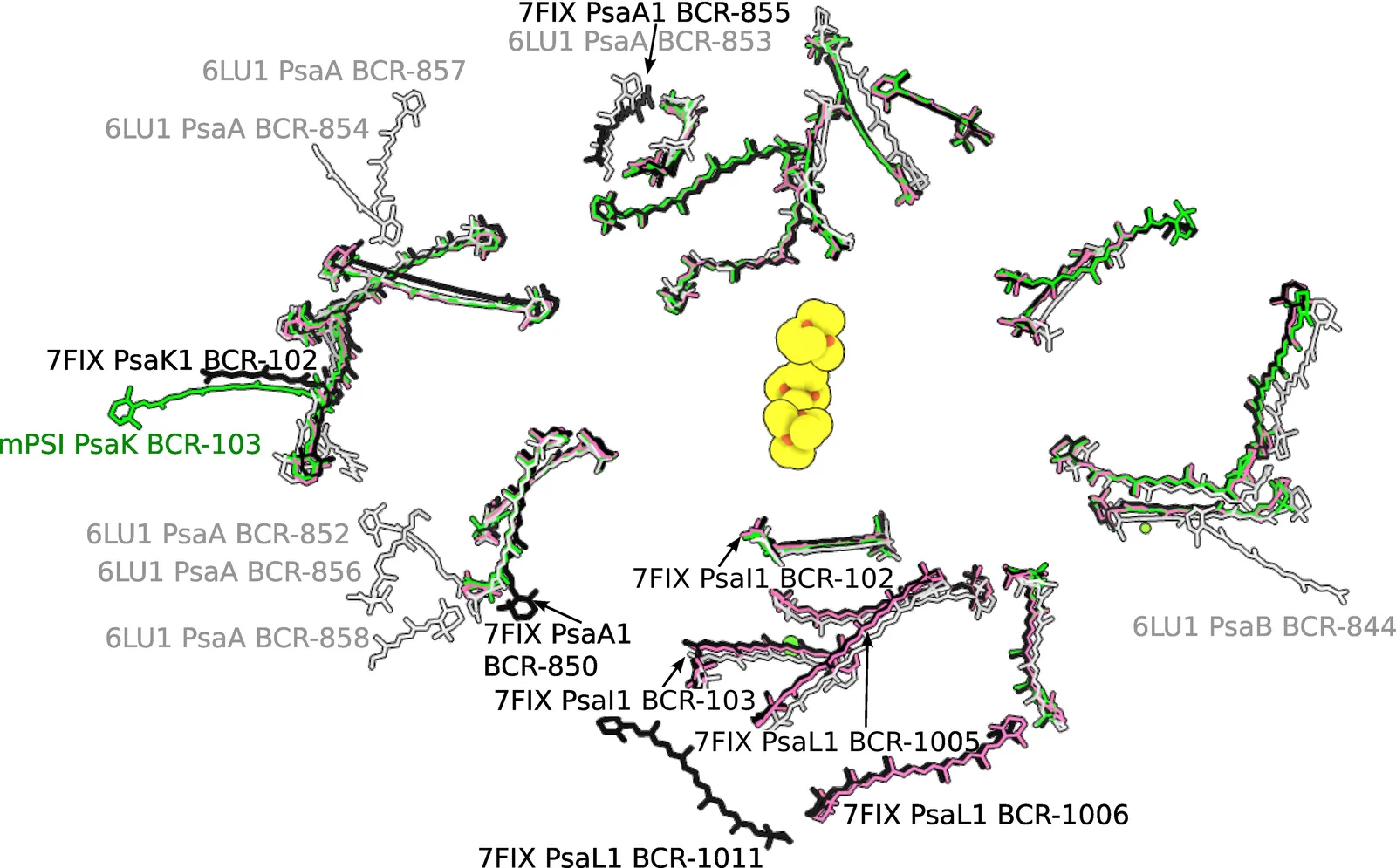
Overlay of carotenoids from different PSI structures. Top view of all ß-Cars in our PSI monomer with the PsaL position at the bottom. FeS clusters coloured in yellow and orange. In comparison to 7FIX (black), 6LU1 (grey) and to 1JB0 (pink), our ßDM mPSI (green) is missing six ß-Cars (7FIX first monomer in black; PsaA: BCR-855; PsaI: BCR-102 and 103; PsaL: BCR-1005, 1006, and 1011)

Most of the ß-Cars (PDB nomenclature: BCR) detected in existing tPSI structures are present in our mPSI structure (Fig. 2). However, compared to the structure of PSI from the same organism with the most ß-Cars found (25 in total in the ßDM tPSI PDB entry 7FIX first monomer (Li et al. 2022)), the following six ß-Cars are missing in the trimerisation region: BCR-855 of PsaA, BCR-102 and 103 of PsaI, and BCR-1005, 1006, 1011 of PsaL. This loss corresponds to 24% of the 25 identified ß-Cars in 7FIX. Furthermore, we were able to model the whole ß-Car structure for BCR-103 of PsaK (7FIX: K-102) and BCR-803 of PsaB (7FIX: B-841) in our mPSI structure. The β-ionone ring of BCR-842 of PsaA (in our mPSI) is oriented away from the PsaL position similar to 1JB0 (BCR-4007), whereas it points towards PsaL in 7FIX (BCR-850). Cofactors and amino acid residues in close vicinity to the ß-Cars are listed in SI, Table S7.

### Lipid and detergent molecules

**Fig. 3:**
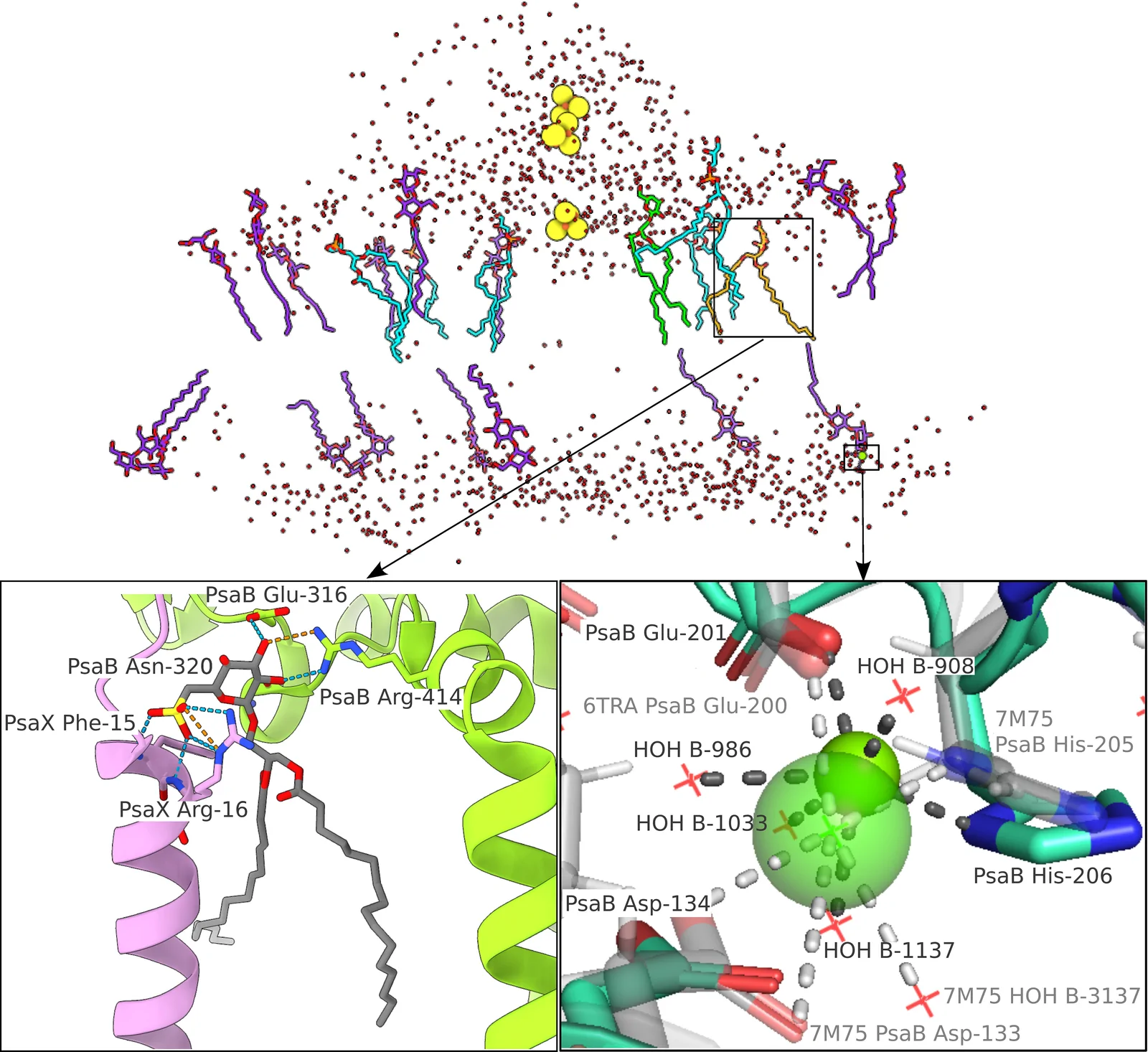
Structural lipids and detergents in the mPSI structure. *Top*: “Pseudo-lipid bilayer” formed by 16 ßDM detergent molecules (violet) and 7 lipids. The lipids are 5 LHG in cyan (compared to 6TRA, instead of PsaM LHG-101 and PsaI LHG-102 almost touching, only one PsaI LHG-101 is present in our structure as well as an additional LHG), 1 MGDG (LMG-804 in green), and 1 SQD (PsaX: SQD-101 in orange; instead of LHG-101 in 7FIX). The red dots are water molecules. *Bottom left*: The newly found SQD (SQD-101) is coordinated by five amino acid residues and one ßDM molecule. *Bottom right*: Coordination of a Mg^2+^ ion at the second Ca^2+^ binding site of *Tv* PSI in our mPSI (greencyan) by four waters (PsaB HOH 1137, 1033, 986, 908) and two amino acid residues of PsaB (Glu-201 and His-206) compared to *Tv t*PSI structure 7M75 (Keable et al. 2021) (grey, semi-transparent) coordinated by Glu-200, His-205 and PsaB HOH 3137. Residue numbers are offset by -1 in PDB 7M75 due to not counting the initiating methionine from reference sequences

We determined seven integral lipid molecules in total in our mPSI structure, consisting of three different lipids found in the thylakoid membrane: five PG molecules (PsaA: LHG-852, LGH-843, LHG-864; PsaX: LHG-104; PsaI: LHG-101), one MGDG (PsaB: LMG-804) and one newly identified SQD molecule for PSI (PsaX: SQD-101) (Fig. 3 and SI, Fig. S10d and S13). Surprisingly, we revealed a large number of detergent molecules with 16 ßDM arranged in a bilayer structure around the peripheral hydrophobic transmembrane region of mPSI (Fig. 3). The upper and lower layers of this structure, referred to as the “pseudo-lipid bilayer”, exhibit comparable dimensions, with a range of 17.6 to 24.3 Å. Moreover, we identified a new SQD molecule (SQD-101), which is coordinated by five amino acid residues (PsaB: Arg 414, Glu 316, Asn 320; PsaX: Phe 15, Arg 16) as well as one detergent molecule (PsaB: LMT-852) (Fig. 3). The coordination of other lipid and detergent molecules in our mPSI structure is listed in SI, Tables S8, S9.

### Water molecules

With a total of 1183 water molecules (SI, Fig. S13), we found the highest number of waters resolved in a mPSI structure. When comparing the water locations of our mPSI to other PSI structures at a distance of 0.5 Å, we find 61% of all waters in 1JB0, 18% in 6TRA, 0% in 6LU1 (only 5 waters are resolved in this structure), 40% in 7FIX and 37% in 5OY0 at similar locations (SI, Table S10). Figure 3 emphasises the antagonistic placement of water at the hydrophilic cytoplasmic (~ 615–635) and lumenal (~ 490) sides compared to the lipids and detergents in the hydrophilic transmembrane region of mPSI. Most water molecules are found outside of the membrane plane (~ 1140), but all Chl *a* molecules are located in the transmembrane region. Nevertheless, there are 40–60 waters (depending on how conservatively we set the membrane borders) present in the membrane area, of which 15 coordinate Mg^2+^ ions of Chl molecules within a distance of 2.0–2.3 Å.

The waters around the P_700_ in our mPSI (9QFP) are similarly located to the ones found in 1JB0 (Jordan et al. 2001) with a difference in-between waters of the two structures of around 0.1 Å probably due to resolution differences. Three waters are located at a distance of 3.4 Å from the P_700_ in both structures respectively (our mPSI: B HOH-939, A HOH-942 and A HOH-1061; 1JB0: B HOH-5005, A HOH-5005, A HOH-5011). Whereas in 9QFP 19 waters are observed within a sphere of 10 Å radius around P_700_, only 9 are observed in 1JB0. Interestingly, both Chl A2 and B2 of the ETC are coordinated each with one water at a distance of 2.1 Å and there are 11 water molecules located between the two PQNs and F_x_. For the tPSI structures with PDB entries 1JB0 and 6TRA, a similar distribution of waters can be observed, but fewer waters are found in the membrane region (1JB0 ~ 35; 5OY0 ~ 40; 6TRA ~ 25) and in-between the PQNs and F_x_ (1JB0 and 5OY0 same 11 H_2_O; 6TRA 7). Of the five waters reported in the mPSI structure PDB 6LU1, three are located in the membrane plane and in-between the PQNs and F_x_.

### Ca^2+^/Mg^2+^ – binding sites within the mPSI structure

In *Tv* tPSI, two Ca^2+^ binding sites were previously discovered in 6TRA (Kölsch et al. 2020) and 7M75 (Keable et al. 2021), while the first Ca^2+^ binding site was already identified in 1JB0 (Jordan et al. 2001). In comparison to these tPSI structures, our mPSI is missing the PsaL subunit including the bound Ca^2+^ and its binding site. However, we find a Mg^2+^ at the location of the second Ca^2+^ binding site at PsaB (Fig. 3). The Mg^2+^ is coordinated by two amino acid residues of PsaB (Glu 201, His 206) and four waters (HOH 1137, 1033, 986, 908). In contrast to tPSI, the Ca^2+^ binding site in our mPSI is now accessible to the surrounding buffer solution, which contains no Ca^2+^, but Mg^2+^ instead. Our mPSI cryo-EM structure revealed an intact second binding site, now occupied by a Mg²⁺ ion, unlike the tPSI structures (Jordan et al. 2001; Malavath et al. 2018; Kölsch et al. 2020; Keable et al. 2021). The distance between this site and the first Ca²⁺ binding site, which is exclusive to ßDM tPSI is around 49 Å.

### Chlorophyll *a*

**Fig. 4:**
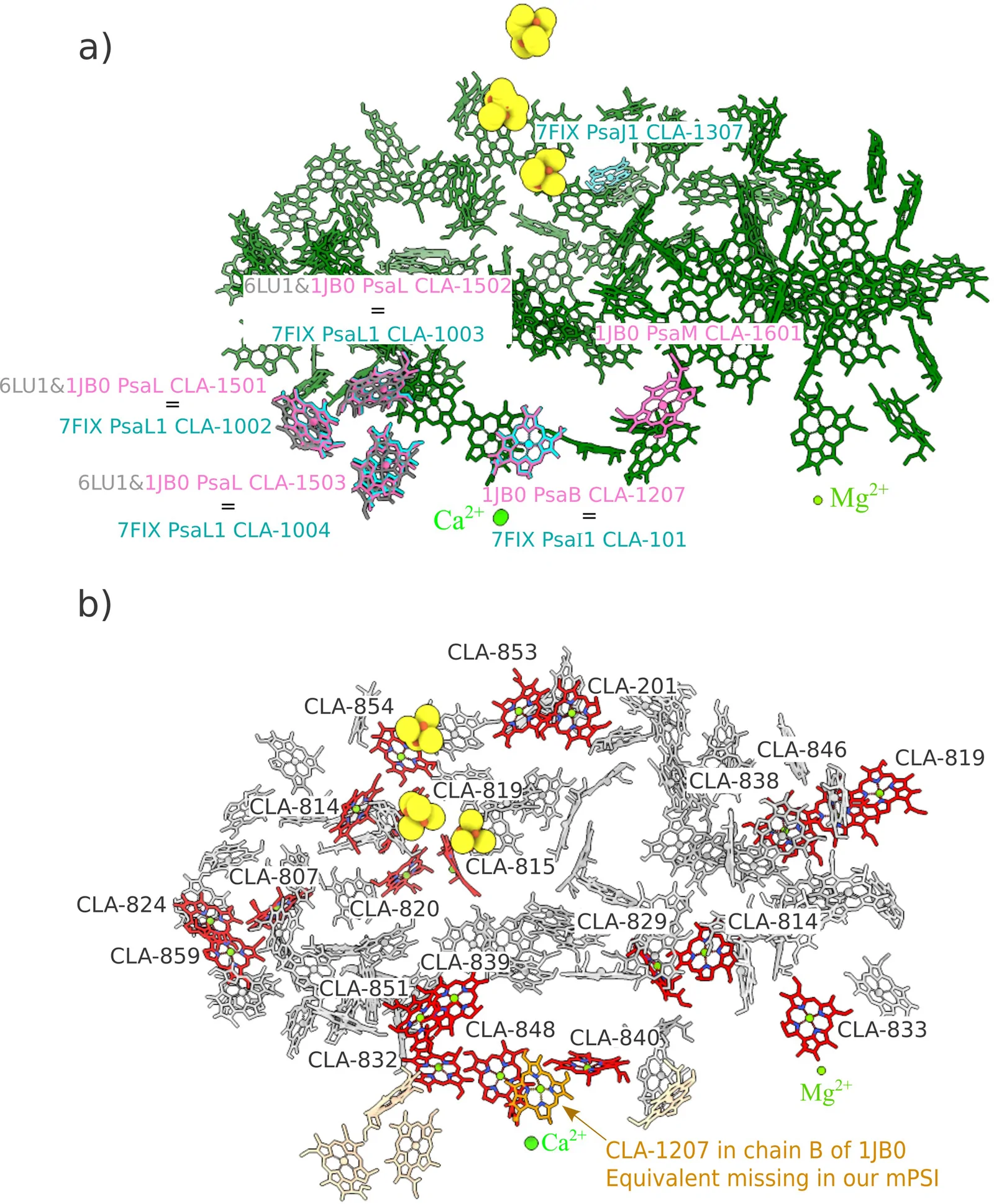
Comparison of Chls in different structures. (**a**) Our mPSI Chls (dark green, no label) shown together with Chls absent in our structure which are coloured and labelled accordingly to the respective tPSI structures 7FIX (cyan), 1JB0 (pink) and the mPSI structure 6LU1 (grey, same residue numbers as in 1JB0 and therefore, residue numbers not listed in grey for CLA-1501-3); phytol tails are omitted for clarity. Also displayed are the Ca^2+^ ion of 1JB0 (light green), Mg^2+^ ion of our mPSI (green), and FeS cluster overlays (orange/yellow). (**b**) Our mPSI (grey) with a monomer from the tPSI structure 1JB0 (Jordan et al. 2001) superimposed. All Chls are shown in stick representation and phytol tails are omitted for clarity. “Red” Chls of our mPSI are coloured in red and labelled with numbers from our mPSI model. All the four “normal” Chls absent in our mPSI but present in 1JB0 are displayed in light orange. The red Chl B-1207 of 1JB0, absent in our structure, is shown in orange

In our mPSI structure, we found 91 Chl *a* molecules; this is five fewer in comparison to tPSI, resulting in a loss of 5.2% of Chl *a*. The missing Chl *a* molecules are: one in PsaM (labelled CLA-1601 in 1JB0, not present in 7FIX), one in PsaJ (labelled CLA-1307 in 7FIX, not present in 1JB0) and one in PsaI, which lies in close proximity to the Ca^2+^ ion as well as the PsaL subunit (labelled CLA-101 in 7FIX and corresponding to CLA-1207 in PsaB in 1JB0) (Fig. 4a) and, not surprising, all three Chl *a* molecules of the missing PsaL subunit (labelled CLA-1501–1503 in 1JB0 and CLA-1002–1004 in 7FIX). In PsaJ, at the outside facing the peripheral part of PSI, the Chl *a* molecules CLA-1306 and 1307 in 7FIX are parallel to each other. In our structure, the former is present (labelled CLA-102), whereas the latter is absent.

### UV-Vis spectroscopy

**Fig. 5:**
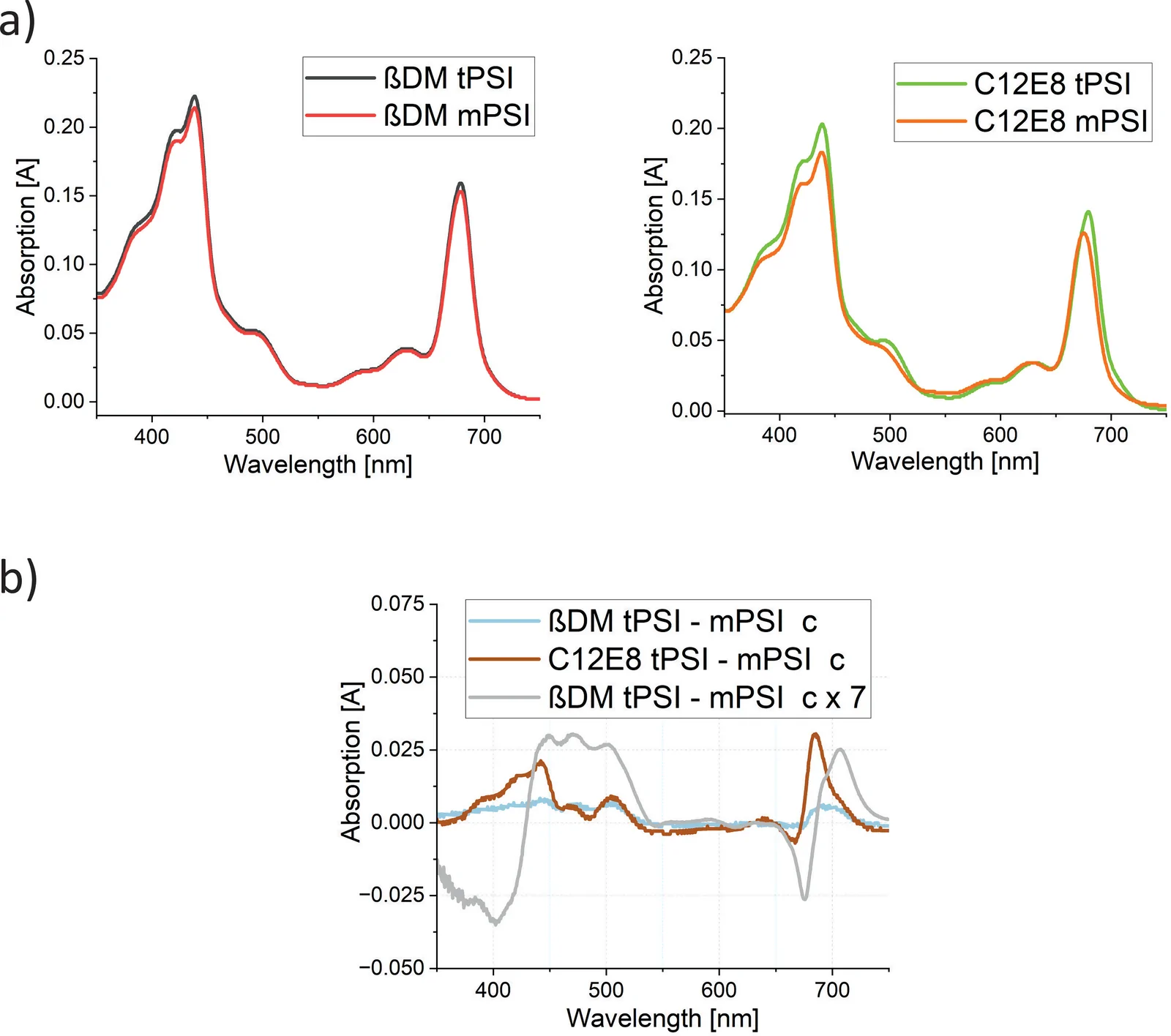
UV-VIS spectra of mPSI compared to tPSI. (**a**) Absorption spectra at RT of tPSI (ßDM in black, C_12_E_8_ in green) and mPSI (ßDM in red, C_12_E_8_ in orange). (**b**) Difference absorption spectra of tPSI minus mPSI (ßDM in light blue, seven-fold higher concentration in grey, C_12_E_8_ in brown). Absorption spectra were normalised to the detergent independent Chl *a* peak at around 630 nm

To analyse the differences of tPSI and mPSI in light absorption, UV-Vis spectra of PSI were measured at room temperature (RT) using either ßDM or C_12_E_8_ PSI. All measurements were normalised to the Chl *a* peak at ~ 630 nm (Fig. 5a), which is less sensitive to environmental factors than the 680 nm peak (Müh and Zouni 2005). To determine the differences in absorption, the trimer-monomer difference spectra were calculated for each detergent (Fig. 5b).

The absorption maximum of tPSI at 680 nm is blue-shifted in mPSI to approximately 678.5 nm (ßDM, G_60_) and 675.5 nm (C_12_E_8_, storage buffer C_30_) (SI, Table S12). Furthermore, the slight shoulder of tPSI at around 708 nm is less pronounced in the ßDM and C_12_E_8_ mPSI samples (Fig. 5a). This together with the blue shift in the absorption spectra of the mPSI samples in this region is hinting to the loss of one or more “red” Chls (Fig. 4b). The tPSI-mPSI difference spectra (Fig. 5b) also reveal a reduction in the absorption peak at 708 nm. This effect is particularly evident in the difference spectrum of C_12_E_8_ tPSI and mPSI (Fig. 5a, right). Additionally, positive bands in the ßDM difference spectrum in the range of 400 to 520 nm could be interpreted as carotenoid bands, confirming the loss of ß-Cars upon monomerisation as suggested by the cryo-EM structure.

In order to interpret the difference spectra, we performed simulations based on Gaussian peaks (SI, Fig. S14). In these simplistic simulations, we only distinguished between “normal” Chls absorbing at 680 nm and two types of “red” Chls absorbing at 710 and 720 nm. In addition, all Chls had a smaller peak at 620 nm. The difference spectra shown in SI, Fig. S14 correspond to the loss of four “normal” Chls and either one 710 or one 720 nm Chl in mPSI compared to tPSI, where the individual absorption spectra are normalised to the 620 nm peak. It is demonstrated that the shape of the measured difference spectra (suggesting band shifts rather than pigment loss) can indeed be rationalised by such a loss of pigments. Interestingly, the zero-crossing of the difference spectrum is more blueshifted (below 700 nm) in the case of a 710 nm Chl being lost compared to the case of a 720 nm Chl. Therefore, it is suggested that the red Chl missing in our mPSI belongs to the pool of red Chls absorbing around 710 nm.

### Fluorescence spectra

**Fig. 6:**
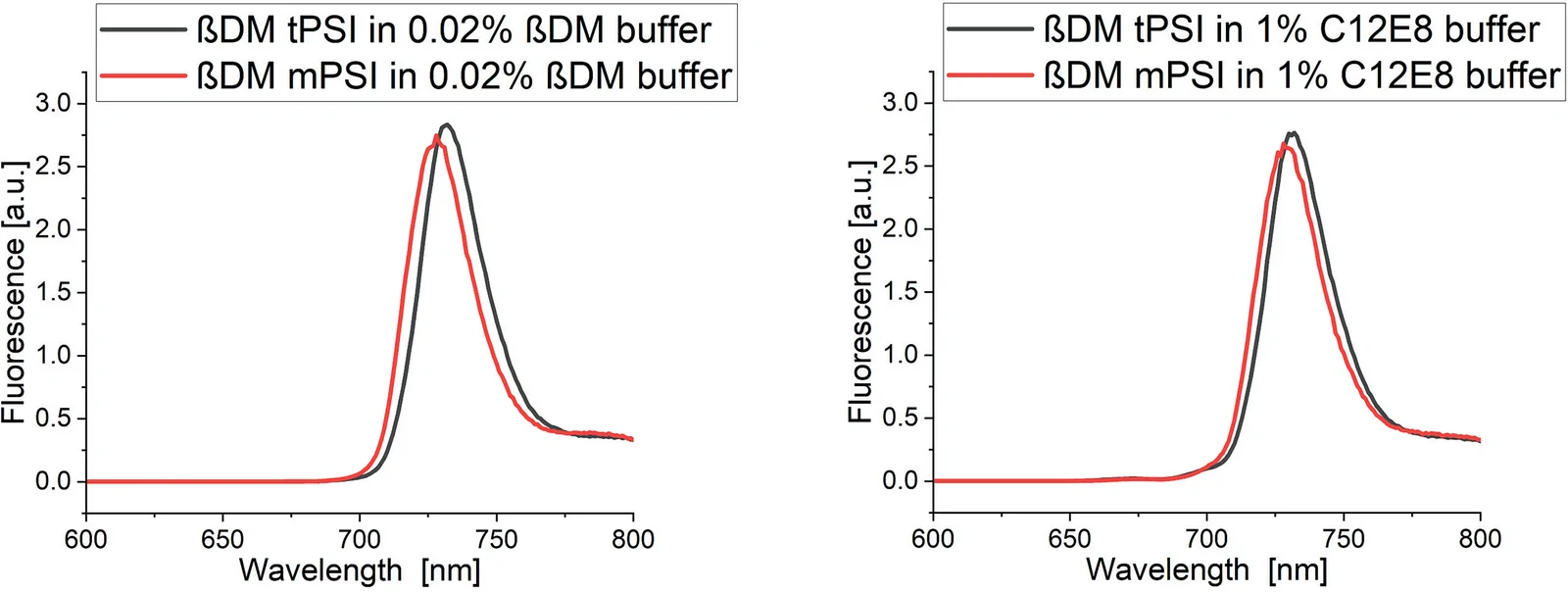
Fluorescence spectra at 77 K of mPSI compared to tPSI. Exemplary 77 K fluorescence emission spectra (excitation at 440 nm) of 120 µg/ml Chl ßDM-purified tPSI (black) and mPSI (red) in buffer containing the detergent ßDM (left) or C_12_E_8_ (right). Fluorescence emission peak values for all tested detergent concentrations in the buffer are listed in SI, Table S12

The position of the fluorescence emission peak gives indications on the nature of the most red shifted Chls in the investigated PSI. However, since the detergent has an influence on the absorption and emission spectra, care has to be taken to avoid misinterpretations (Müh and Zouni 2008). Therefore, we focus on the analysis of emission peak maxima on samples with a rather high protein concentration and, consequently, a higher protein/detergent ratio. In these cases, the emission maxima are shifted from 731 to 732 nm in tPSI to 727–730 nm in mPSI (Fig. 6 and SI, Table S12). This rather moderate blue shift is much smaller than that observed by Çoruh et al. (2021) in their monomer preparation, showing a peak maximum at 720 nm. We therefore suggest that a different type of red Chl is missing in our monomer preparation compared to that of Çoruh et al. (2021).

### Thermal stability

CD measurements were conducted to gain insights into the thermal stability of PSI in monomeric and trimeric form in buffer with different detergent content (SI, Table S13). In general, all mPSI samples are similarly or less stable than the corresponding tPSI. The ßDM-purified samples (mPSI, tPSI) in 0.02% ßDM buffer as well as in 1% ßDM exhibit similar transition temperatures (*T*_transition_) above 80 °C. Compared to that, ßDM- and C_12_E_8_-purified tPSI in 1% C_12_E_8_ buffer show a lower *T*_transition_ around 57–59 °C. In buffer with 0.013% C_12_E_8_, *T*_transition_ values of the C_12_E_8_-purified PSI samples differ by less than 10 °C with tPSI being more stable than mPSI. The stability of C_12_E_8_ tPSI drops, when using 1% C_12_E_8_ in the buffer, whereas the stability of the C_12_E_8_-purified mPSI increases significantly to > 65 °C, when measuring it in buffer containing 1% ßDM, resulting in a T_transition_ similar to C_12_E_8_-purified tPSI in buffer with 0.013% C_12_E_8_. Finally, the highest transition temperatures of > 90 °C were determined for C_12_E_8_- as well as ßDM-purified tPSI with 1% ßDM in the buffer.

### Binding of Cyt *c*_6_ to mPSI

**Fig. 7:**
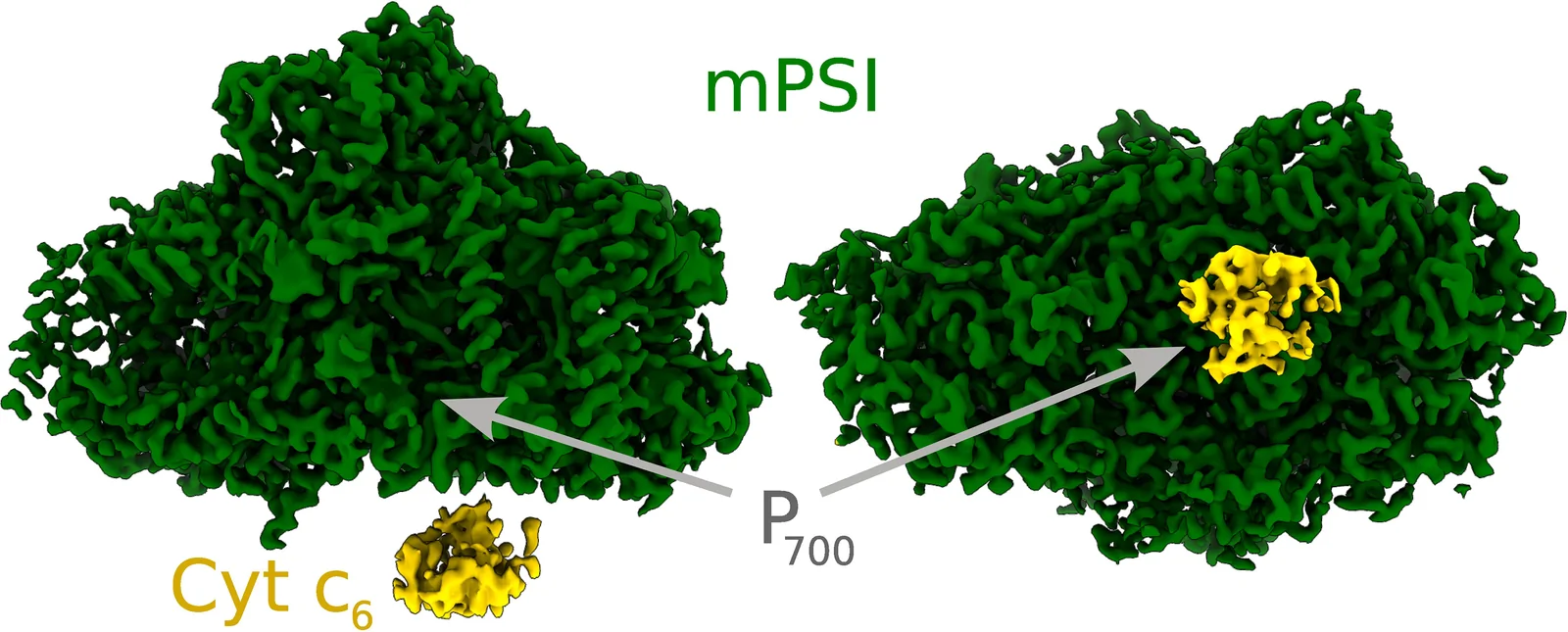
Potential binding site of Cyt *c*_6_ (yellow) at mPSI (green). The cryo-EM analysis (contour level 0.066) of reduced mPSI after adding Cyt c_6_ reveals additional density in the vicinity of P_700_, which we attribute to Cyt *c*_6_. *Left*: side view from membrane plane (PsaI, M in front); *Right*: bottom view from the lumen (PsaI, M on top)

When adding the native *Tv* Cyt *c*_6_ to our mPSI under reducing conditions, we find an additional density in the cryo-EM map, located at the luminal side of mPSI (Fig. 7). The global resolution of this reconstruction is 2.01 Å, with poorer local resolution (5 to 8 Å) at the density attributable to Cyt *c*_6_. This density did not allow model building and could not be improved by local refinement, likely due to the small size of Cyt *c*_6_ (10 kDa) and to its high mobility. Low occupancy of Cyt *c*_6_ is consistent with its weak binding affinity for tPSI.

### Solution structures of mPSI and of the mPSI-Cyt *c*_6_/mPSI-Cyt c_HH_ complex

In order to investigate the solution structure of the mPSI complex at physiological temperature, we performed small angle neutron scattering (SANS) experiments. The use of specifically deuterated βDM (dßDM) detergent suppressed the signal arising from free βDM micelles in solution (SI, Fig. S6A), so that the scattering contribution from micelles was eliminated. At the same time, the R_g_ value obtained from the Guinier analysis, 50.37 ± 0.08 Å, and the D_max_ value estimated with Gnom program, 155 ± 10 Å, were considerably larger than the corresponding values calculated from the cryo-EM structure of mPSI presented here, 40.2 Å and 131 Å, respectively. Further details of the model-independent analysis are provided in SI Table S2. This discrepancy suggests that the detergent exchange was not complete and more strongly bound βDM is at least partly retained within the detergent belt.

SI, Fig. S6C shows a fit of the SANS data using a structural model of the mPSI–βDM complex based on the cryo-EM structure obtained in this study. The excellent fit quality demonstrates that the high-resolution cryo-EM structure also describes mPSI in solution state at physiological temperature. Next, we considered the binding of cytochrome to mPSI. SI, Fig. S6B shows SANS data for mPSI–dβDM (red line) and the mPSI–dβDM–Cyt *c*_HH_ complex (blue line). As visible in the inset of SI, Fig. S6B, the data for the complex with Cyt *c*_HH_ are shifted towards lower Q values, indicating a larger complex than in the absence of cytochrome. This finding, as well as slightly increased values of R_g_ and D_max_ (50.42 ± 0.19 Å and 160 ± 10 Å, respectively), indicates binding of Cyt *c*_HH_. A structural model of the mPSI–dβDM–Cyt *c*_HH_ complex based on the cryo-EM structure obtained in this study is shown in SI, Fig. S6D.

## Discussion

### Monomeric PSI

There exist many resolved *Tv* tPSI structures (Jordan et al. 2001; Li et al. 2022; Kölsch et al. 2020), but only one *Tv* mPSI structure (Çoruh et al. 2021) (PDB 6LU1; a comparison can be found in SI, Table S14), at a resolution of 3.2 Å and in which the PsaL subunit and its Chl *a* molecules are preserved, but the PsaK and PsaX subunits are missing. In that structure, the PsaF and PsaJ subunit sequences could only be modelled at around 20% in relation to the tPSI X-ray structures 1JB0 and 6TRA. In addition, in 6LU1 compared to 1JB0, 14 Chl *a*, one lipid (LHG-5004) and four Cars (each one in PsaB, PsaF, PsaJ and PsaL) are missing; this breaks the cluster symmetry proposed by Jordan et al. (2001). The lower global resolution of the mPSI structure 6LU1 (3.2 Å) for peripherally located co-factors is not sufficient to explain this loss.

Here in this study, we present a novel and efficient protocol to convert purified ßDM tPSI into active mPSI at high yield, using only an increase in temperature and the addition of the detergent C_12_E_8_. Our mPSI solution is homogeneous with particles having a hydrodynamic radius (R_h_) of R_h_ = 45.3 ± 0.7 Å (SI, Table S1). This value fits well to the radius of gyration (R_g_) of 41 Å calculated from the crystal structure 1JB0 and the slightly higher values for R_g_ = 58 Å and R_h_ = 69 Å in Golub et al. (2017). In our mPSI cryo-EM structure (mPSI with detergent belt in SI, Fig. S7), all subunits are present as in the PSI trimer, except for the PsaL subunit. The PsaL subunits are located at the centre of the trimerisation region of tPSI and the monomerisation of tPSI is causing the PsaL subunit to dissociate, as previously suggested (Chitnis and Chitnis 1993; Domonkos et al. 2004; Kłodawska et al. 2015).

The percentage of absent Cars (24%) in our structure is exceeding the loss of Chls (5.2%) by a factor of more than 4.5 and, therefore, the changes of Car absorption should be more evident than the absorption differences due to Chl loss. All missing Chl *a* molecules except for the one in PsaJ are located closely together around the missing PsaL subunit. It is reasonable that the loss of these cofactors including the Ca^2+^ ion is an accompanying effect of the detachment of PsaL.

Our samples show a blue-shift of the main absorption peak of mPSI (ßDM 678.5 nm, C_12_E_8_ 675.5 nm) in comparison to tPSI (680 nm) for both detergents (Fig. 5). Interestingly, the blue shift is three times larger for the C_12_E_8_ purified than for the ßDM purified samples. In addition, the difference absorption spectrum (tPSI minus mPSI) indicates a clear loss of Cars in the range of 400–550 nm which is consistent with earlier results (Karapetyan et al. 2014; El-Mohsnawy 2019).

### Comparison of B-factors in mPSI vs. tPSI ETC

A direct comparison of the B-factors between different structures is not informative, as it mostly reports on differences in resolution between the structures. Hence, a more informative comparison is the difference in B-factors between different locations within the same structure: The magnitude of this local difference can then be compared between different structures. In all of the tPSI structures analysed here (SI, Fig. S11), the A-branch of the ETC exhibits higher B-factors and is therefore more flexible compared to the more rigid B-branch. This is likely the result of its radial arrangement in the trimer, where the B-branch always faces inwards to the centre of the trimer and the A-branch is located more radially outside. In our mPSI structure, the B-factors are similar for both branches and this is also true for the mPSI structure from 6LU1 (Çoruh et al. 2021). Moreover, all analysed structures of monomeric and trimeric PSI (SI, Fig. S11) show an increase in flexibility of the iron sulphur clusters from the inside of PSI to the outside, with F_B_ being the most flexible. Implications of this are longer distances to coordinating residues (Tyr-735 3.1 Å, Tyr-743 3.6 Å) or even missing coordination (to Tyr-603 and Thr-743). In addition, the distances in the ETC of our mPSI are more similar to the trimeric 1JB0 and 6TRA than to the monomeric 6LU1.

### Mg^2+^ at the Ca²⁺ binding sites in PSI

Most tPSI structures show one Ca²⁺ binding site per monomer, which is localised at the luminal loop between the first and second transmembrane helices of PsaL, PsaA and the PsaL of the neighbouring monomer (Jordan et al. 2001; Malavath et al. 2018). In addition, a second Ca^2+^ binding site is found at the luminal side of PsaB (Mazor et al. 2015, 2017; Malavath et al. 2018; Kölsch et al. 2020; Keable et al. 2021). Surprisingly, we identified a Mg²⁺ ion at the second Ca²⁺ binding site. Besides the differences in waters, the only absent coordination, compared to 7M75 (Keable et al. 2021), is the one of Asp-134 (Asp-133 in 7M75) which can be explained by the discrepancy in distances between Ca^2+^ and Asp-133 (2.6 Å) in 7M75 and between Mg^2+^ and Asp-134 (4.2 Å) in our mPSI structure. In the *Synechocystis* ßDM tPSI structure 5OY0, the second Ca²⁺ binding site is located approximately 58 Å away from the first Ca²⁺ binding site, close to PsaB. This suggests that the original Ca²⁺ ion binds non-specifically or weakly to the PsaB subunit and can easily be replaced by Mg²⁺ ions present in the buffer containing no Ca^2+^ when this binding site is exposed to the buffer in mPSI. We assume that the second binding site is regulated by an auxiliary protein on the luminal surface of PSI. However, the role of the second Ca²⁺ binding site remains unclear (Malavath et al. 2018) and we were not able to identify any calcium ions in the region of the missing PsaL subunit in our ßDM mPSI structure. This contrasts with the ßDM tPSI X-ray structure 1JB0 (Jordan et al. 2001), which identified Ca²⁺ ions in this region and retained the PsaL subunit. However, the cryo-EM ßDM mPSI structure 6LU1 (Çoruh et al. 2021) could not localise any Ca²⁺ ions due to an undefined loop region of the PsaL subunit, even though the PsaL was still attached.

### Transient interaction between mPSI and Cyt *c*_6_

When adding the native *Tv* Cyt *c*_6_ under reduced conditions, we detected a density at the luminal side of mPSI near P_700_, where Cyt *c*_6_ is expected to bind (Fig. 7). Hence, we attributed this density to bound Cyt *c*_6_. Despite a high global resolution of 2.01 Å, the binding of Cyt *c*_6_ to PSI is not structurally resolvable likely due to the dynamics of the transient binding process, as was already observed previously at an even higher global resolution of 1.97 Å (Li et al. 2022). The non-native structure determination of crosslinked monomeric algal PSI:Cyt *c*_6_ was recently achieved for *Chlamydomonas* (Ogawa et al. 2026) and is therefore a possible objective for *T. vestitus* PSI.

Our SANS data show a binding of horse heart Cyt *c*_HH_ to mPSI, but could not detect binding for the native Cyt *c*_6_. This finding confirms results of previous SANS experiments, where binding of Cyt *c*_HH_ – but not Cyt *c*_6_ – to tPSI was achieved (Kölsch et al. 2020). The different behaviour of the two cytochromes was attributed to the transient interaction combined with a higher binding affinity of the non-native Cyt *c*_HH_ to tPSI (*K*_M_ ≈ 5–45 µM) compared to Cyt *c*_6_ (*K*_M_ ≈ 30–300 µM). In the present study, we confirmed the latter effect for mPSI and observed a mPSI-Cyt *c*_HH_ complex in solution at RT using SANS and provide a structural model of the latter complex based on the cryo-EM structure obtained here (SI, Fig. S6D).

### Low energy Chls

The difference absorption spectrum (tPSI minus mPSI) indicates a loss of Chls absorbing above 700 nm. Although these spectra show features reminiscent of spectral shifts, our simplistic simulations show that their shape is consistent with a loss of Chls, including red Chls (Fig. S14). Up to date, the role of the red Chls is not understood and their exact position in the tPSI structure is debated (see the image of suggested red Chls, Fig. 4b). Possible functions suggested, are protection at high light intensities or energy collection at low light intensities (Byrdin et al. 2002; Damjanović et al. 2002; Sener et al. 2002; Zazubovich et al. 2002; Schlodder et al. 2007; Akhtar et al. 2021; Sohail et al. 2025). In an exciton model of red Chls, Vaitekonis et al. (2005) have theoretically modelled the excitation energy spectrum of *Tv* tPSI and on the basis of the structure 1JB0 (Chl *a* residue names) assigned one Chl tetramer (A31-A32-B6-B7), three trimers (B31-B32-B33, A2-A3-A4, A19-A20-A21), one dimer (A38-A39) and one monomer (B11) (comparison to our mPSI in SI, Table S11). Furthermore, the red Chl absorption bands at 708, 715 (Zazubovich et al. 2002), and 719 nm could be identified previously at low temperatures (4 K) where energy transfer between the Chls is limited (van Grondelle et al. 1994). Our preliminary modelling of the absorption difference spectra suggests that it is not one of the most red shifted Chls that is missing in our mPSI preparation. This suggestion is in agreement with the rather moderate blue shift of the low-temperature emission maximum detected in fluorescence, indicating that red Chls absorbing at 719 nm are likely still present. Our results, therefore, suggest a loss of Chls contributing to the 708–710 nm absorption band.

Interestingly, in our mPSI cryo-EM structure, we are missing only the red Chl labelled “B7” (B-1207) in 1JB0, which is close to the position of the missing PsaL. It is part of the red Chl tetramer A31-A32-B6-B7, which Vaitekonis et al. (2005) calculated to have the lowest energy state in tPSI and rather interpret it as a strongly coupled dimer (A31-A32) with associated monomers on each side. Different studies designated these red Chls also as a strongly coupled Chl trimer A31(A832)-A32(A848)-B7 responsible for the 719 nm spectral peak (Byrdin et al. 2002; Damjanović et al. 2002; Sener et al. 2002; Zazubovich et al. 2002; Vaitekonis et al. 2005; Schlodder et al. 2007; Akhtar et al. 2021).

Since the detergent influences the Chls, one has to be careful when performing fluorescence experiments. To minimise detergent effects (see, e.g., (Müh and Zouni 2008)), we therefore investigated the fluorescence at 77 K at high protein concentrations. Firstly, the shapes of all spectra (Fig. 6) indicate the high purity of our samples (mPSI and tPSI, both in each detergent). Secondly, we find a blue shift in fluorescence emission for mPSI compared to tPSI as well. This blueshift is rather small and could be due to the detergent perturbing the red Chls. Possibly, the Chls giving rise to the ~ 730 nm emission are located at the monomer-monomer interface in tPSI and, hence, are more prone to detergent effects in mPSI. A loss of Chls absorbing at 720 nm should – in our view – cause a much larger blue shift similar to the one observed in 6LU1. In the latter, providing the only other existing structure of monomeric *Tv* PSI (Çoruh et al. 2021), not only B33 (B-1233) of the red Chls suggested by Vaitekonis et al. (2005) is missing, but also 13 other Chls. On the other hand, engineering an additional “red” Chl in trimeric PSI from *Synechocysti*s at a homologous position resulted in a red shift of only 2 nm (from 722 nm to 724 nm) in 77 K fluorescence emission spectra (Toporik et al. 2020). Therefore, an assignment of specific Chls to the 720 nm pool remains difficult. In contrast, our results clearly suggest that the Chls missing in our mPSI preparation do not belong to this pool or are not relevant for the far-red emission. Rather, one of them, which is possibly B-1207 (B7), is involved in the absorption at 708 nm.

### Role of detergents and lipids

Notably, an exact quantification of the detergent in a membrane protein solution could not yet be accomplished and all the literature up to date is plagued by this limitation. Membrane proteins from the thylakoid membrane are known to be delipidated by detergent treatment, leading to a non-native conformational change of a PDC and as a result, the activity of the PDC decreases (Guskov et al. 2009; Kern and Guskov 2011; Brady et al. 2022). Therefore, styrene-maleic acid lipid particles (SMALPs) and other membrane mimetic systems (MMs) are now often used to avoid detergents and enable a more native conformation of the protein through a native-like lipid bilayer environment (Popot 2010; Brady et al. 2019). According to Brady et al. (2022), a MM containing the *Tv* tPSI-styrene-maleic acid lipid particle (tPSI-SMALP) complex was shown to have significantly faster energy transfer and charge separation rates 1000 times faster than an isolated βDM tPSI complex (Cherepanov et al. 2020; Brady et al. 2022). Based on lipidomics analysis, approximately eleven lipid molecules per monomer were found for the βDM tPSI particles, including four SQDs. Moreover, with approximately 1,300 lipids per tPSI, the SMALP-tPSI particles contain 44 times more lipids than βDM PSI particles and 100 times more than the 1JB0 crystal structure.

In *Tv* PSI, the anionic lipids PG and SQD are found only in pockets containing positively charged residues, mainly Lys and Arg, as was shown for PSII in the X-ray structure of dimeric photosystem II in ßDM (ßDM dPSII) (Guskov et al. 2009). During solubilisation and purification with detergent, delipidation occurs at the hydrophobic surface of the protein. Galactolipids (MGDG and DGDG) in and around the protein are thought to be exchanged due to their structural similarity to the detergent βDM (Guskov et al. 2009; Kern et al. 2009; Brady et al. 2022). On the other hand, C_12_E_8_ molecules have not yet been detected in the C_12_E_8_ dPSII structure, which is possibly due to their flexibility (Hellmich et al. 2014).

In total, we identified seven endogenous lipids in our βDM-mPSI structure: five PG, one MGDG, and one SQD. Additionally, we detected 16 βDM detergent molecules arranged in a bilayer around the hydrophobic transmembrane region (Fig. 3). Hence, our mPSI structure has 23 lipid locations. This is comparable to the 17 lipids per monomer found in a *Synechocystis* sp. PCC6803 βDM PSI trimer X-ray structure (PDB entry 5OY0) with 51 lipids in total (27 PG, 16 MGDG, seven SQD and one DGDG), of which 37 lipids were located within the PSI core and the remaining 14 at the periphery of the trimer (Malavath et al. 2018). Moreover, we found the same MGDG (LMG) as in 1JB0 and two of the three PG (LHG) molecules assigned in 1JB0 and 7FIX. Additionally, we resolved three more PGs (LHG X-104 (close to the SQD), A-864, I-101) compared to 1JB0 and two more than in 6TRA (X-104, A-864). The latter has a PG (LHG M-101) at the position of our PG (LHG I-101) and another PG (LHG I-102) located in close proximity (~ 7 Å, at some positions, less than 5 Å) to M-101 (in 6TRA, I-101 of our structure).

Our *Tv* mPSI structure is the first to identify a SQD (SQD-101) instead of the third PG (LHG B-5004) found in 1JB0, 7FIX (LHG X1-101) and in 6TRA (LHG B-851) at the same position. This might be due to the high resolution of our ßDM mPSI structure combined with a careful preparation. The finding fits well to experiments creating a mutant unable to produce SQD, where the PG content substantially increased (Endo et al. 2016). In contrast, no lipid is present at the position of our SQD in 6LU1. Therefore, the latter mPSI structure has only three integral lipids in total. Similar to the seven SQD found in the *Synechocystis* structure 5OY0 (Malavath et al. 2018), our SQD is also located on the stromal side of PSI. It is noteworthy that a SQD has been localised near the PsaL subunit in 5OY0 (Malavath et al. 2018), since a monomerisation of tPSI was observed in the absence of SQD in a mutant with inhibited SQD synthesis (Endo et al. 2016). Remarkably, the SQD content in SMALP tPSI is about 50% higher than in thylakoid membranes. Furthermore, it was hypothesised that dynamic oligomerisation is promoted by electrostatic repulsion between anionic lipids, which in turn makes the lipid bilayer more fluid (Brady et al. 2022). Therefore, we hypothesise that the number of SQD molecules not only in the thylakoid membrane but also at the monomer-monomer interface of tPSI and in PsaL, determines the different oligomeric forms of PSI.

The nearly complete dissociation process of tPSI into mPSI at 60 °C was inspired by recent circular dichroism (CD) measurements on *Tv* ßDM tPSI (Golub and Boyka et al. 2025). Accordingly, the addition of the non-ionic detergent C_12_E_8_ significantly reduces the PDCs thermal stability, from an initial temperature of approximately 80 °C to 60 °C (see Fig. 9; Table 4 in (Golub and Boyka et al. 2025)). In this study, we show that CD transition temperatures decrease for each PSI sample upon addition of 1% C_12_E_8_ and in contrast, show the highest stability with additional 1% ßDM in the buffer. Remarkable is also the increase of transition temperature (~10 °C) for C_12_E_8_ mPSI when using 1% ßDM in the buffer. Notably, the thermal stability of mPSI is reduced to a similar extent as tPSI in ßDM or C_12_E_8_ (*T*_transition_ ~55–60 °C) and the PDCs with 0.02% ßDM of tPSI and mPSI also have a comparable *T*_transition_ value at ~80–90 °C (SI, Table S13). The CD data are consistent with small-angle X-ray scattering measurements (SAXS) at RT on ßDM tPSI (Golub and Boyka et al. 2025), which showed that tPSI has a ßDM belt around the protein 5.2 times larger than C_12_E_8_. The ßDM belt is composed of stiffer molecules and should therefore be more stable than a belt containing the narrower and more flexible C_12_E_8_ molecules (Golub and Boyka et al. 2025). In summary, the flexibility of C_12_E_8_ appears to destabilise mPSI in accordance with the detergent dependent decrease (C_12_E_8_) and increase (ßDM) in thermal stability of the PDC.

Furthermore, on the basis of our structural findings, we hypothesise that during the membrane solubilisation, the detergent molecules are not only surrounding tPSI in a belt, but are also moving into the PSI structure and extract some lipids as well as could change the positions of or extract Chls residing in the outer parts in close contact with the detergent belt. C_12_E_8_ is more flexible and has a slimmer head group than ßDM, so that it can better enter the space in between the monomers in the trimer and hence may enable the monomerisation. Furthermore, the X-ray structure of *Synechocystis* ßDM PSI 5OY0 (Malavath et al. 2018) reveals a lipid bilayer at the monomer-monomer interface in tPSI, which is believed to contribute to the stabilisation of the trimer. Additionally, in a cryo-EM structure of the trimeric *Tv* βDM PSI-ferredoxin complex at 1.97 Å resolution (pdb 7FIX; (Li et al. 2022)), 17 new lipid acyl chains for each monomer–monomer interface were modelled. These lipids could be responsible for the stabilisation of the trimeric form of PSI. Due to their high flexibility, these could play a crucial role in the monomerisation of the PSI trimer (Li et al. 2022). Hence, we suspect that the formation of mPSI from tPSI comprising either the C_12_E_8_ detergent or the C_12_E_8_-ßDM mixture, occurs due to decreased stability of the PDC as a result of partial delipidation via the highly flexible detergent C_12_E_8_, most likely at the monomer-monomer interface of tPSI. The flexibility of C_12_E_8_ molecules in and around the tPSI complex is enhanced by increasing the temperature (30 to 60 °C) and hence improves the monomerisation process and therefore, results in an increased mPSI yield (SI, Fig. S1).

## Conclusions and outlook

Our reproducible preparation process facilitated the monomerisation of tPSI *via* enhanced lipid exchange at the monomer-monomer interface by detergents and temperature, which allowed us to generate the highest resolution of a monomeric PSI structure to date and to identify the density of bound Cyt *c*_6_ close to P_700_ in mPSI. In addition, we could detect SQD for the first time in *Tv* PSI as well as a Mg^2+^ at the second Ca^2+^ binding site and relate the red Chl B7 to the absorption peak at 708 nm. Moreover, our protocol for the creation of mPSI provides the framework for several subsequent experiments, such as the structure determination via cryo-EM of a C_12_E_8_ purified mPSI to investigate detergent-dependent structural changes in PSI, and the study of the red Chls at the monomer-monomer interface by enabling their accessibility in a native monomeric PSI. Additionally, a SMALP cryo-EM structure should be produced to determine the structure of PSI together with its native lipids without any detergent present. Further investigation of the structural positions of the red Chls could be realised by modelling approaches in combination with mutations (single Chl addition or knock-out) in relation to resulting spectra and red Chl positions in the structure. Furthermore, after using optimal binding conditions regarding ions and pH of the buffer determined by Kölsch et al. (2020), the binding affinity of Cyt *c*_6_ to PSI can be enhanced by introducing mutations to Cyt *c*_6_. Our findings establish a robust basis for the reliable generation of mPSI, which will enable the separation of 3D classifications of mPSI with bound Cyt *c*_6_, and are therefore expected to facilitate the identification of the binding site of native non-crosslinked *Tv* Cyt *c*_6_ in the near future.

## Experimental procedures

### Isolation, purity, and characterisation of trimeric PSI

Trimeric PSI of *Thermosynechoccocus vestitus (Tv) BP1* was isolated and purified as previously described (Golub and Boyka et al. 2025). The tPSI was crystallised in either G_0_ buffer (5 mM MES, pH 6; 0.02% ßDM) adjusted to a final conductivity of 0.5–0.7 mS / cm or at a Chl concentration *c*_Chl *a*_ = 10 mM and mixed 1:50 with C_0_ (5mM MES, pH 6; 0.013% C_12_E_8_). After crystallisation, tPSI samples were diluted in either G_60_ (5 mM MES, pH 6, 60 mM MgSO_4_, 0.02% ßDM) or C_30_ (5 mM MES pH 6; 30 mM MgSO_4_; 0.013% C_12_E_8_). Sample purity was confirmed via polyacrylamide gel electrophoresis (PAGE). For blue native (BN) PAGE, a polyacrylamide gradient from 3 to 8.5% was used (after (Wittig et al. 2006)), while for SDS-PAGE according to Laemmli (1970), layers of 12% and 15% of the running gel were cast.

Intact protein masses of PSI subunits < 20 kDa were determined by matrix-assisted laser desorption/ionisation time-of-flight mass spectrometry (MALDI-TOF MS). Spectra were acquired on an ultrafleXtreme MALDI-TOF/TOF instrument (Bruker Daltonics, Bremen, Germany) equipped with a Smartbeam-II solid-state laser operating at 2000 Hz. Samples were prepared using the dried-droplet method. Protein samples were mixed 1:1 (v/v) with a sinapinic acid matrix solution (10 mg/ml) prepared in TA33 solvent (33% acetonitrile, 67% water, 0.1% trifluoroacetic acid) and spotted onto a ground steel target plate. Mass spectra were recorded in positive-ion linear mode over a *m/z* range of 2,000–20,000. External calibration was performed using Protein Calibration Standard I (Bruker Daltonics). For each sample spot, 500 laser shots were accumulated per spectrum, and multiple spectra were recorded and averaged to obtain a representative mass spectrum. Data acquisition and processing were carried out using FlexControl and FlexAnalysis software (Bruker Daltonics). Measurements were performed for PSI samples in either C_12_E_8_ and ßDM (mPSI *n* = 4 and *n* > 10; tPSI *n* = 4 and *n* > 10 from Golub and Boyka et al. (2025).

tPSI was further characterised by size exclusion chromatography using a Superose™ 6 Prep Grade (17-0489-01, GE Healthcare Bio-Sciences, Uppsala) column (d = 1 cm, h = 30 cm, CV = 23.56 ml) at a constant flow rate of 0.5–1 ml / min. The column was equilibrated (3–5 CV) and eluted (2–3 CV) with either G_60_ for ßDM PSI or C_30_ for C_12_E_8_ PSI. Prior to application to the column, each sample was filtered through a 0.2 μm filter (Puradisc 30/0.2 CA S, Whatman). Subsequently, 0.5 ml of each sample were loaded onto the column and the capillary loop was emptied with 1 ml of the respective buffer. The C_12_E_8_ samples eluted earlier from the column compared to samples in ßDM buffer.

The sample homogeneity of mPSI and tPSI was determined via dynamic light scattering (DLS) employing a DynaPro NanoStar (Wyatt technology Corp.; software DYNAMICS™ 7.1.9.3) at 787 nm in DLS buffer (25 mM Tricine-NaOH, pH 8.0, 50 mM NaCl and either 0.02% ßDM or 0.013% C_12_E_8_) at 20 °C after filtering the sample through a 0.22 μm syringe driven filter unit (low protein binding Durapore (PVDF) membrane for fine particle removal from aqueous and mild organic solutions, Cat. NO. SLGVR04NL, Millex^®^ - GV, Japan) in 4 µl-disposable MicroCuvettes (Wyatt technology). All used DLS measurements exhibit Pd-values below 15% indicating a homogeneous solution.

A Clark-type electrode (Oxygraph^+^, Hansatech Instruments, Germany; software O_2_ view 2.05, 2013) was used to measure the PSI activity via oxygen (O_2_) reduction (oxygen consumption) at 20 °C in oxygen buffer (25 mM Tricine-NaOH, pH 8.0, 0.2 mg/ml sodium ascorbate, 0.05 mg/ml methyl viologen, either 0.02% ßDM or 0.013% C_12_E_8_ and 16 µM cyt c_HH_) under constant mixing at 60 rpm and excitation via illumination of > 500 µmol_Photons_ / (m² • s) in triplicate.

### Cyt *c*_6_ purification

The used Cyt *c*_6_ contained a C-terminal 6xHis-tag and was expressed heterologously in *Escherichia coli*, isolated and purified as previously described (Kölsch et al. 2018).

### Monomerisation of tPSI to mPSI

Both pre-crystallised C_12_E_8_ and ßDM tPSI samples were used for subsequent monomerisation. Different temperatures, incubation times and percentages of C_12_E_8_ were tested. With ßDM-purified tPSI, the highest yield of ~ 90% mPSI was achieved at 60 °C for 30 min, 1% of C_12_E_8_. tPSI purified with C_12_E_8_ needed to be incubated only for 10 min at 60 °C for similar results. Only about ~ 10% mPSI was produced from the tPSI when βDM tPSI was incubated at 60 °C for 30 min without C_12_E_8_.

After the monomerisation procedure described above, the PSI solution was loaded onto a sucrose gradient with 10–35% (w/v) sucrose in sucrose buffer (20 mM MES, pH 6.0; 30 mM MgSO_4_ and either 0.02% ßDM or 0.013% C_12_E_8_) and the separation was performed using a L-70 Ultracentrifuge (Beckman, Brea) at 30,000 rpm, 4 °C for 15 h. The distinct green bands were pooled according to their density and concentrated in a centrifugal concentrator (Vivaspin^®^ 20, 100 kDa MWCO, Sartorius) or Amicon^®^ (Millipore) with a 100 kDa MWCO polyethersulfone (PES) membrane (Biomax^®^, Millipore).

### UV/Vis, CD, and fluorescence spectroscopy

UV/Vis spectroscopy was conducted with a SPECORD^®^ 200 PLUS (Analytik Jena, Edition 2010) in 1 cm quartz cuvettes (Hellma^®^) for comparison of the spectra of tPSI and mPSI in G_60_ and C_30_. For concentration determination either G_60_ or 80% acetone were used together with an extinction coefficient of Chl *a* of ε_680, ßDM_ = 57.1 / (mM • cm) at 680 nm for ßDM solution (Müh and Zouni 2005) or in 80% acetone ε_664, C12E8_ = 76.8 / (mM • cm) at 664 nm for C_12_E_8_, respectively (Porra et al. 1989).

CD spectroscopy was performed with a J-815 JASCO spectropolarimeter to determine the melting temperature *T*_*m*_ of the unfolding reaction. CD spectra were recorded in a 1 mm quartz cuvette (polarimetric, 110-1-P-40, Hellma) in the range of 200–250 nm at a scanning speed of 50 nm/min, accumulation of 3 scans and a band width of 0.5–1 nm, at 0.1–0.2 g/l Chl *a* concentration. The temperature was increased via a Peltier thermostated cell holder (PTC-423 S, JASCO) from 20 °C to 95 °C for ßDM PSI and for C_12_E_8_ PSI samples to 80 °C. For CD measurements, the used buffers were either G_60_ or C_30_. The Jasco Spectra Manager 2 software was used for measurement as well as export of the collected data.

Under the assumption of a two-state reversible equilibrium between the native (*F*) and denaturated states (*U*), the fraction of denaturated protein, *f*_*U*_, at temperature, *T*, was identified using Eq. (1) with *Θ*_*T,222*_ being the ellipticity observed at 222 nm and temperature *T*. The ellipticities at 222 nm of fully folded (*Θ*_*F*_) and unfolded (*Θ*_*U*_) states were measured at 20 °C and 95 °C in the case of ßDM and 80 °C for samples with C_12_E_8_, respectively.

1 $$\:U{f}_{U}\:=\:\frac{{\varTheta\:}_{T,\:222}\:-\:{\varTheta\:}_{F}}{{\varTheta\:}_{U}\:-\:{\varTheta\:}_{F}},$$

The nonlinear Boltzmann Eq. (2) of OriginPro, Version 2023 (OriginLab Corporation, Northampton, MA, USA),

2 $$\:y\:=\:{A}_{2}\:+\:\frac{{A}_{1}\:-\:{A}_{2}}{1\:+\:{e}^{\frac{x\:-\:{x}_{0}}{dx}}}$$

was deployed for analysing the measured denaturation curve to derive *T*_*m*_ (= *x*_0_), using the initial (*A*_1_ = *Θ*_*F*_) and final (*A*_2_ = *Θ*_U_) CD plateau signals and the slope (*dx*) of the curve.

Fluorescence emission spectra were obtained with a Horiba Scientific Fluoromax+ spectrophotometer at 77 K with a slit width of 3 nm using an excitation wavelength of λ_ex_ = 440 nm with a slit width of 3 nm and recording emission spectra between 600 and 800 nm with a slit width of 3 nm. tPSI and mPSI samples were diluted approximately 30 s before freezing to achieve a final Chl *a* concentration of 2.5 µg/ml in either G_60_ (with the total ßDM concentration of either 0.02%, 0.5% or 1%) or C_30_ (with the total C_12_E_8_ concentration of either 0.013%, 0.5% or 1% C_12_E_8_) buffer. 0.5 ml of sample was flash-frozen in liquid nitrogen immediately before measurement. The data was normalised to the total emission.

### Small-angle neutron scattering (SANS) sample preparation, experiments and data analysis

SANS experiments were performed using the small angle instrument Quokka at the Australian Nuclear Science and Technology Organization ANSTO in Lucas Heights (Australia). In order to suppress detergent scattering, protiated β-dodecyl maltoside detergent (hβDM) was exchanged for partially deuterated β-dodecyl maltoside (dβDM) provided by the National Deuteration Facility of ANSTO in Lucas Heights, Australia, according to previously reported procedures (Midtgaard et al. 2018). The detergent exchange was performed by size-exclusion chromatography following the procedure used by Midtgaard et al. (2018), which was also applied by Golub et al. (2022). The dβDM was characterized by NMR and electrospray ionization mass spectrometry, giving an overall deuteration level of approximately 77% and an alkyl-chain deuteration level of approximately 89%. The overall deuteration level was calculated from the mass-spectrometric isotope distribution using DGet! Software (Lockwood and Angeloski 2024). The samples were adjusted to a protein concentration of about 5 mg/ml, loaded into 2 mm thick round quartz cells and measured on Quokka (Wood et al. 2018) with 6 Å neutrons at two configurations: one with a sample to detector distance of 10 m and a source to sample distance of 10 m as well as a second with a sample to detector distance of 2 m and a source to sample distance of 4 m. Samples were measured for two hours at the longer sample to detector distance and one hour at the shorter distance. Data were reduced using Igor Pro macros (Kline 2006) using standard procedures and placed on an absolute scale using the direct beam measurement through a calibrated attenuator.

The pair distance distribution function P(R) and the maximum interparticle distance *D*_max_ were determined using the Indirect Fourier Transform (IFT) method employing the software routine GNOM (Svergun 1992). The IFT analysis was carried out in a limited Q-range of up to 0.12 Å^−1^, where the influence of the background was low.

Models of the mPSI-detergent complex were constructed using the MP Builder plugin (Molodenskiy et al. 2021) to PYMOL (PyMOL, Version 0.99) (DeLano 2006). The resulting pdb-code was fitted to the SANS data using PEPSI (Grudinin et al. 2017).

### Cryo-EM specimen preparation and screening

Prior to cryo-EM sample preparation, the ßDM-C_12_E_8_ mPSI sample was rebuffered in the ßDM-containing buffer G_60_. Quantifoil R 1.2/1.3 300 Cu grids were glow-discharged in a Pelco easiGlow (20 mA, 60 s, 0.4 mbar of residual ambient air, negative polarity) before application of 4 µL of mPSI at 2.5 mM Chl *a* on the carbon film side. The plunge-freezing was performed with a Vitrobot Mark IV device, with the chamber at 4 °C and 100% relative humidity, 30 s of incubation time between application and blotting, 2 s of blotting (at blot force 0) before vitrification in liquid ethane.

The mPSI-Cyt *c*_6_ complex was formed in darkness by addition of sodium ascorbate to a final concentration of 5 mM to the mPSI solution and addition of Cyt *c*_6_ at a 100-fold molar excess over mPSI. All handling from complex formation to vitrification was performed in darkness to minimise dissociation of the complex.

Grids were screened on a Glacios microscope operated at 200 kV to identify a suitable grid for high-resolution data collection.

### Cryo-EM data collection of mPSI

A total of 22 001 movies were collected on a Titan Krios G3 operated at 300 kV and equipped with a Gatan K3 BioQuantum detector. The nominal magnification of 130 000x resulted in a raw pixel size of 0.648 Å/px. The energy filter slit width was 20 eV. The total dose was 40 e/Å^2^ and fractionated into 40 movie frames. The nominal defocus range was -1.4 to -0.4 μm by steps of 0.2 μm.

### Cryo-EM data collection of mPSI with Cyt *c*_6_

A total of 13 236 movies were collected on a Titan Krios G3 operated at 300 kV and equipped with a TFS Falcon4i detector and Selectris energy filter. The nominal magnification of 165 000x resulted in a raw pixel size of 0.734 Å/px. The energy filter slit width was 10 eV. The total dose was 40 e/Å^2^ and fractionated into 40 movie frames. The nominal defocus range was -1.6 to -0.4 μm by steps of 0.2 μm.

### Cryo-EM image processing of mPSI

Image processing was performed with CryoSPARC version 4.5 (Punjani et al. 2017). A summary of the processing strategy is shown in Supplementary Fig. S9. Raw movies were motion corrected using the Patch Motion Correction job with default parameters. CTF parameters were estimated using the Patch CTF estimation job with default parameters. The resulting micrographs were grouped based on the aberration-free image shift (AFIS) groups from data collection. 1628 micrographs (7% of the total) had a CTF fit resolution worse than 10 Å and were excluded from further processing. 1010 particles were manually picked from a random subset of 100 micrographs to produce a training set for Topaz (Bepler et al. 2019). A topaz picking model was trained using the ResNet8 architecture, a micrograph downscaling factor of 10 and an expected number of particles per field of view of 150. Picking the 20 336 micrographs resulted in 1 000 224 particles, subsequently extracted in a box 448 px wide, Fourier cropped to 64 px, and submitted to reference-free 2D classification (100 classes, circular mask diameter 180 Å). 945 000 particles were retained based on features of the corresponding 2D class averages. These particles were sorted by *ab initio* reconstruction followed by heterogeneous refinement (8 classes). 753 759 particles from good classes were retained, extracted in a box 448 px wide, Fourier cropped to 128 px and subjected to a new round of heterogeneous refinement (4 classes). A homogeneous refinement performed with all good particles (713 596 particles) reached 2.64 Å resolution. 678 particles were identified as duplicates (based on pick coordinates closer than 20 Å after 3D refinement) and excluded from further analysis. The 2.64 Å reconstruction was used to perform reference-based motion correction (estimating hyperparameters and empirical dose weights) of the entire particle stack, which excluded 152 particles (by excluding micrographs containing too few particles for trajectory fitting) and re-extracted the remaining 712 866 motion-corrected particles at the raw pixel size (0.648 Å) in a box 512 px wide. A final 3D homogeneous refinement was performed with the following settings: non-uniform regularisation (Punjani et al. 2020), minimise over per-particle scale, optimise per-particle defocus, optimise per-group CTF parameters (tilt and trefoil), Ewald sphere curvature correction (negative curvature, determined empirically by testing both curvatures). This final refinement reached 1.78 Å resolution, and the map was sharpened with B = -35.8 Å^2^ determined by a Guinier fit. Data collection and image processing statistics are reported in SI, Table S3.

### Cryo-EM image processing of mPSI with Cyt *c*_6_

The same image processing strategy as for mPSI was applied to the mPSI-Cyt *c*_*6*_ dataset. This yielded a reconstruction at 2.01 Å resolution from 59 062 particles, in which no additional density could be detected compared to the mPSI reconstruction. Masked 3D classification following a procedure similar to that described for the crosslinked PSI: Cyt *c*_6_ complex from *Chlamydomonas* (Ogawa et al. 2026) identified 8 571 particles producing a reconstruction at 2.81 Å resolution in which density consistent with Cyt *c*_6_ was observed. This density did not allow model building, and local refinement could not improve it.

### Atomic model building and refinement

An atomic model was automatically built into the 3D reconstruction using ModelAngelo version 1.0.12 (Jamali et al. 2024) (with no prior sequence information, build_no_seq). The resulting profile hidden Markov models (HMM) were searched against the reference proteome of *Thermosynechococcus vestitus* BP1 (Uniprot accession code UP000000440). The following proteins were identified with high confidence (low E-value): PsaA, PsaB, PsaF, PsaD, PsaC, PsaE, PsaJ, PsaI, PsaK, PsaM and p4.8 K (SI, Table S4).

AlphaFold2 predictions of all these proteins were obtained from AlphaFold-DB (Jumper et al. 2021; Varadi et al. 2022). The predicted models were aligned to the automatically built model using the matchmaker command in ChimeraX version 1.8 (Pettersen et al. 2021). This helped to correctly place PsaA and PsaB that share a high sequence identity and an identical fold, and were therefore difficult to assign visually. All models were rigidly fit into the map using the fitmap command and combined as multiple chains into a single model. Protein termini not supported by density were trimmed, and co-factors were copied from PDB entry 1JB0 (Jordan et al. 2001). The model was then exhaustively inspected interactively in ISOLDE (Croll 2018) and corrected where necessary. Automated refinement was performed with servalcat version 0.4.88 (Yamashita et al. 2021), and the model was further corrected using the sharpened map and difference map produced by servalcat as MDFF target and visual guides in ISOLDE. A bound ion was confirmed to be Mg^2+^ from considering the ions present in the sample, refining different ions and comparing their refined B-factors to those of the surrounding protein residues, and using the CheckMyMetal server (Zheng et al. 2014). A final refinement was performed with servalcat (riding hydrogens and isotropic B-factors, 10 refinement cycles). The reconstruction was analysed with OccuPy version 0.1.13 (Forsberg et al. 2023) to determine its relative scale and feature occupancies (the kernel settings were: input lowpass 8.0 Å, kernel radius 6.17 pixels, kernel size 9 pixels, tau percentile 99.29%, tile size 12 pixels).

Validation statistics on the map and model, and agreement between the model and map were computed using phenix.validation_cryoem from the Phenix suite version 1.21.2–5419 (Liebschner et al. 2019). Atomic model refinement and validation statistics are shown in SI, Table S3.

## Supplementary Information

Below is the link to the electronic supplementary material.

Supplementary Material 1

## Data Availability

The authors declare that the data supporting the findings of this study are available within the paper and its Supplementary Information files. Should any raw data files be needed in another format they are available from the corresponding authors upon reasonable request. For the mPSI structure, the cryo-EM maps and masks were deposited in the EMDB with accession code EMD-53120, the atomic model was deposited in the PDB with accession code 9QFP, and the raw cryo-EM movies were deposited to EMPIAR with accession code EMPIAR-12814. For the mPSI-Cyt c6 reconstruction, the cryo-EM maps and masks were deposited in the EMDB with accession code EMD-57205, and the raw cryo-EM movies were deposited to EMPIAR with accession code EMPIAR-13422.
