# Supplementary material for: High-resolution structure of monomeric photosystem I from Thermosynechococcus vestitus
Source: Photosynth Res. 2026 Oct 15;164(6):52. doi: 10.1007/s11120-026-01237-x (PMC13672387; doi:10.1007/s11120-026-01237-x)
Supplement: Supplementary file 1 [file 11120_2026_1237_MOESM1_ESM.pdf]

Supporting Information

High-resolution structure of monomeric Photosystem I from *Thermosynechococcus vestitus*

J. Boyka[a], G. Gaullier[b], J. Forsman[c,h], A. T. Graça[c, i], M. Golub[d], N. Croy[b], K. Wood[e], A. P. Duff[f], M. Moir[f], J. Pieper[d], J. Messinger[b,c], F. Müh[g*], A. Zouni[a*] and C. Blikstad[b]*

[a] *Department of Biology, Humboldt-Universität zu Berlin, 10099 Berlin, Germany*

[b] *Department of Chemistry - Ångström Laboratory, Uppsala University, Uppsala 75120, Sweden*

[c] *Umeå Plant Science Centre, Department of Plant Physiology, Umeå University, 90817 Umeå, Sweden*

[d] *Institute of Physics, University of Tartu, 50411 Tartu, Estonia*

[e] *Australian Centre for Neutron Scattering, Australian Nuclear Science and Technology Organization, Lucas Heights, NSW 2234, Australia*

[f] *National Deuteration Facility, Australian Nuclear Science and Technology Organization, Lucas Heights, NSW 2234, Australia*

[g] *Department of Theoretical Biophysics, Institute for Theoretical Physics, Johannes Kepler University Linz, 4040 Linz, Austria*

[h] *Current Address: Department of Biochemistry, University of Otago, 9016 Dunedin, New Zealand.*

[i] *Current Address: European Molecular Biology Laboratory (EMBL), EMBL Grenoble, 38042 Grenoble, France.*

*Author to whom correspondence should be addressed:
C. Blikstad: cecilia.blikstad@kemi.uu.se
F. Müh: frank.mueh@jku.at
A. Zouni: athina.zouni@hu-berlin.de

Generation of functional monomeric PSI

We tested different temperatures, incubation times, and detergent concentrations and carried out a control experiment in which only the detergent ßDM was present in the tPSI sample during incubation (Fig. S1). The $C_{12}E_8$ tPSI and mixed detergent ßDM-$C_{12}E_8$ tPSI complex were directly dissociated into monomers by temperature treatment at 60 °C, yielding approximately 90% of mPSI (see section 'Experimental procedures'). Additionally, we demonstrated that the more stable ßDM tPSI samples without any $C_{12}E_8$ detergent form only 10% monomers at 60 °C (Fig. S1). After monomerisation, the separation of mPSI from tPSI was performed on a sucrose gradient and by ultracentrifugation (Fig. S2).

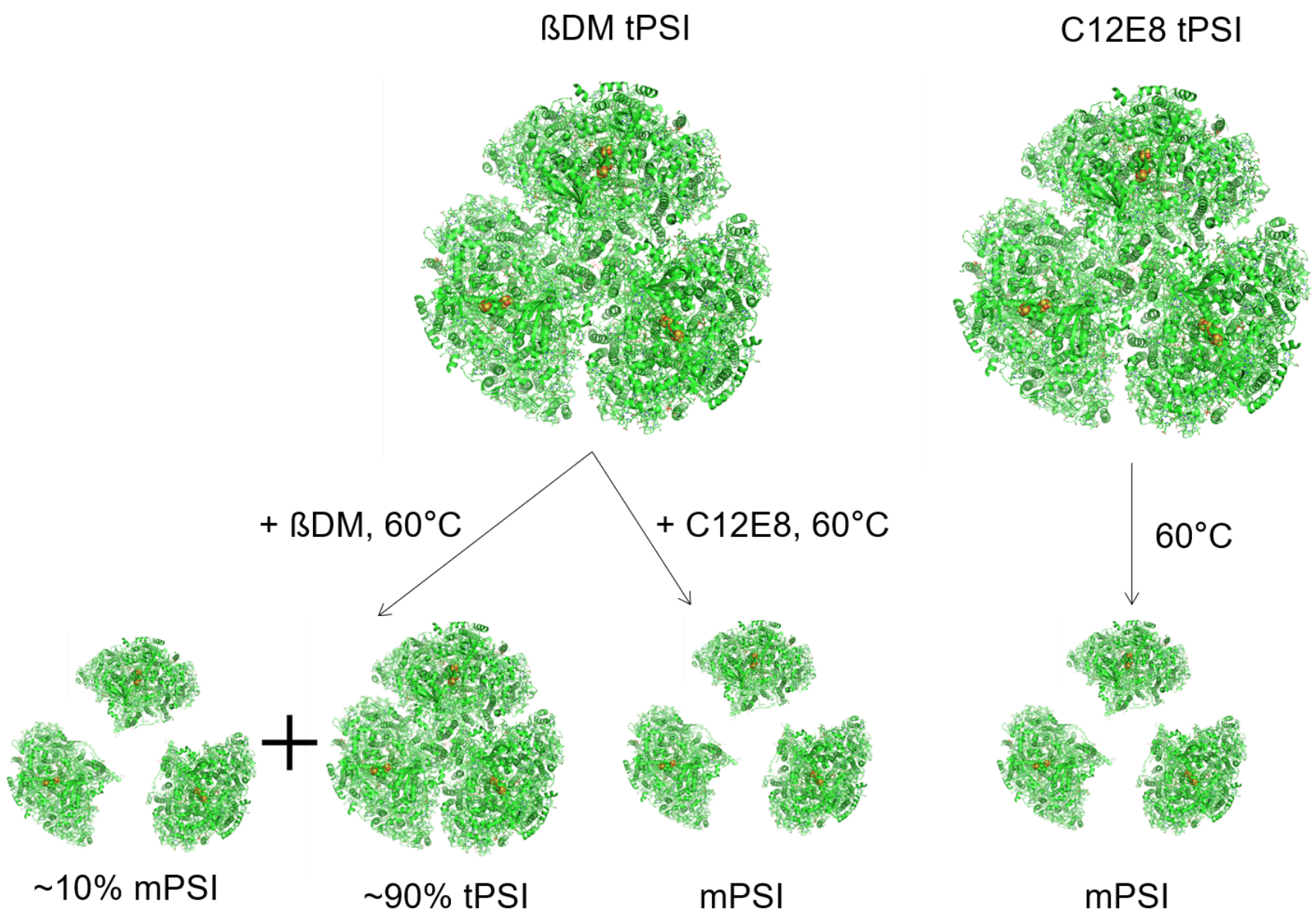

Fig. S1 Scheme of monomerisation of PSI due to addition of $C_{12}E_8$. Only when the detergent $C_{12}E_8$ is added, is tPSI, purified in either ßDM (top left) or $C_{12}E_8$ (top right), converted to a high amount to mPSI after incubation at 60 °C. Just a very small fraction of mPSI is formed, when only ßDM is present.

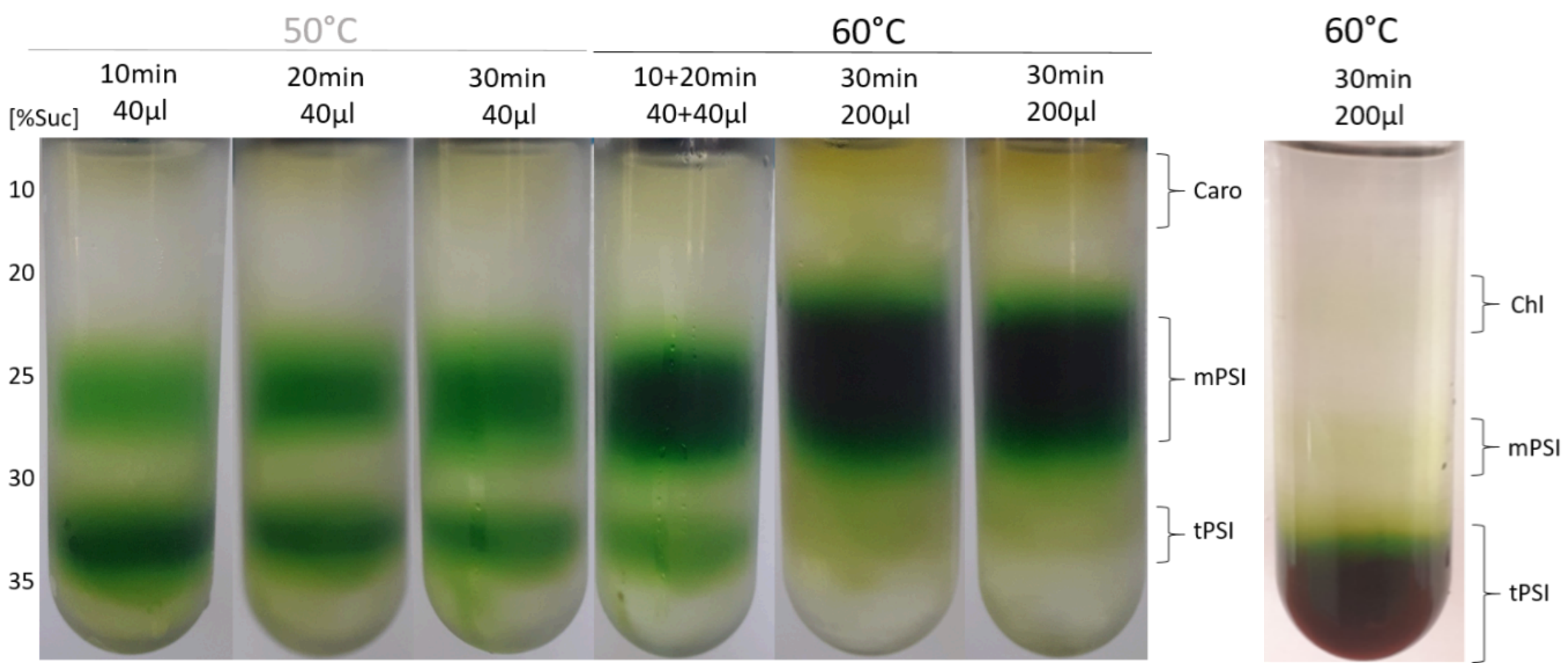

Fig. S2 Sucrose gradients. Different proportions of mPSI and tPSI are obtained for different incubation times and temperatures during the monomerisation procedure with ßDM purified PSI and added $C_{12}E_8$ (volumes loaded onto the sucrose gradient are indicated in µl). The control experiment (right) with only the detergent ßDM shows almost exclusively intact trimeric PSI.

Subsequently, the mPSI bands were pooled, concentrated and the buffer exchanged to G_{60} (ßDM) or C_{30} ($C_{12}E_8$). The purity and homogeneity of the samples was ensured by performing Blue-Native (BN) and SDS gels, MALDI-ToF (Fig. S4) as well as DLS. BN gels exhibit a single band for tPSI and mPSI with either ßDM or $C_{12}E_8$, indicating high purity of the samples. The SDS gels confirm the presence of the bigger subunits, PsaA and PsaB, which run around ~ 55 - 70 kDa. MALDI-ToF spectra show the detected other ten subunits for tPSI (PsaL, D, F, C, K, E, J, I, X, M) and for mPSI all but PsaL.

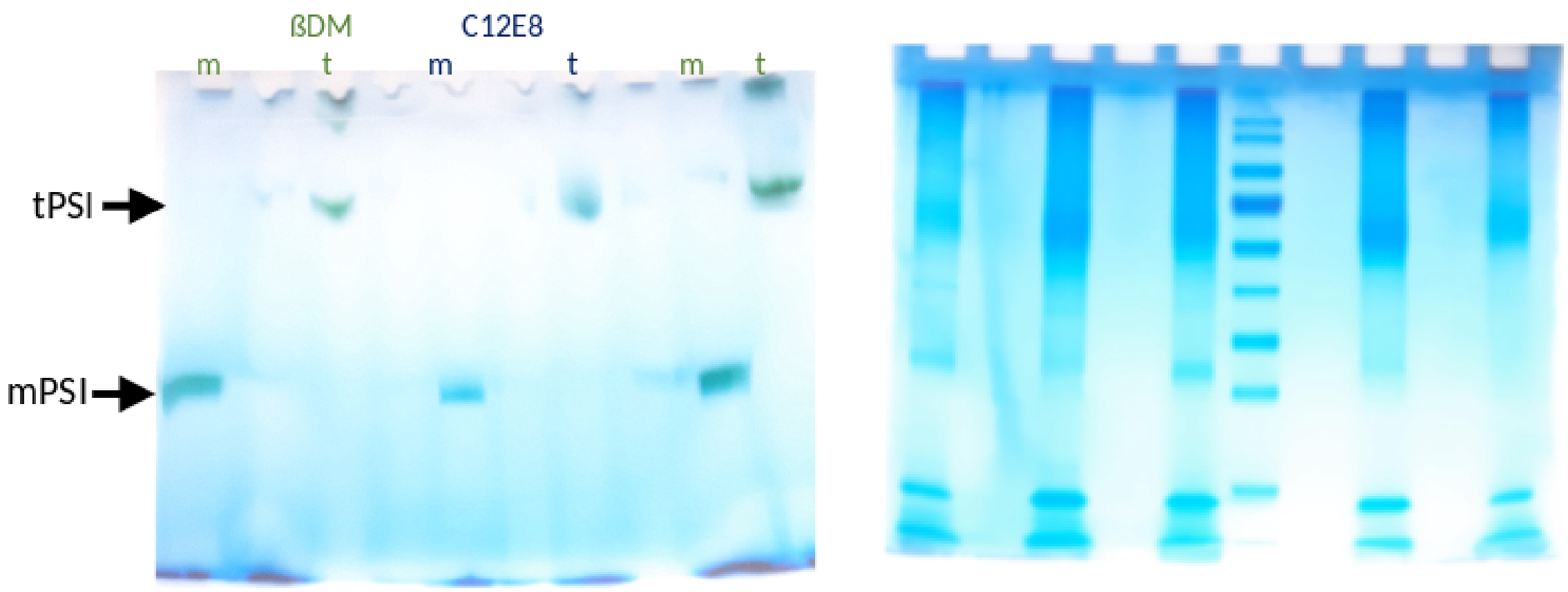

Fig. S3 Gel electrophoresis. *Left*: BN-PAGE demonstrates the high purity of tPSI (parts of this gel were already published (Golub and Boyka et al. 2025) and mPSI samples. *Right*: SDS-PAGE indicates the presence of the core subunits (PsaA and PsaB) together with bands at around 15 kDa and 10 kDa corresponding to PsaL, D, F and PsaC, K, E, respectively.

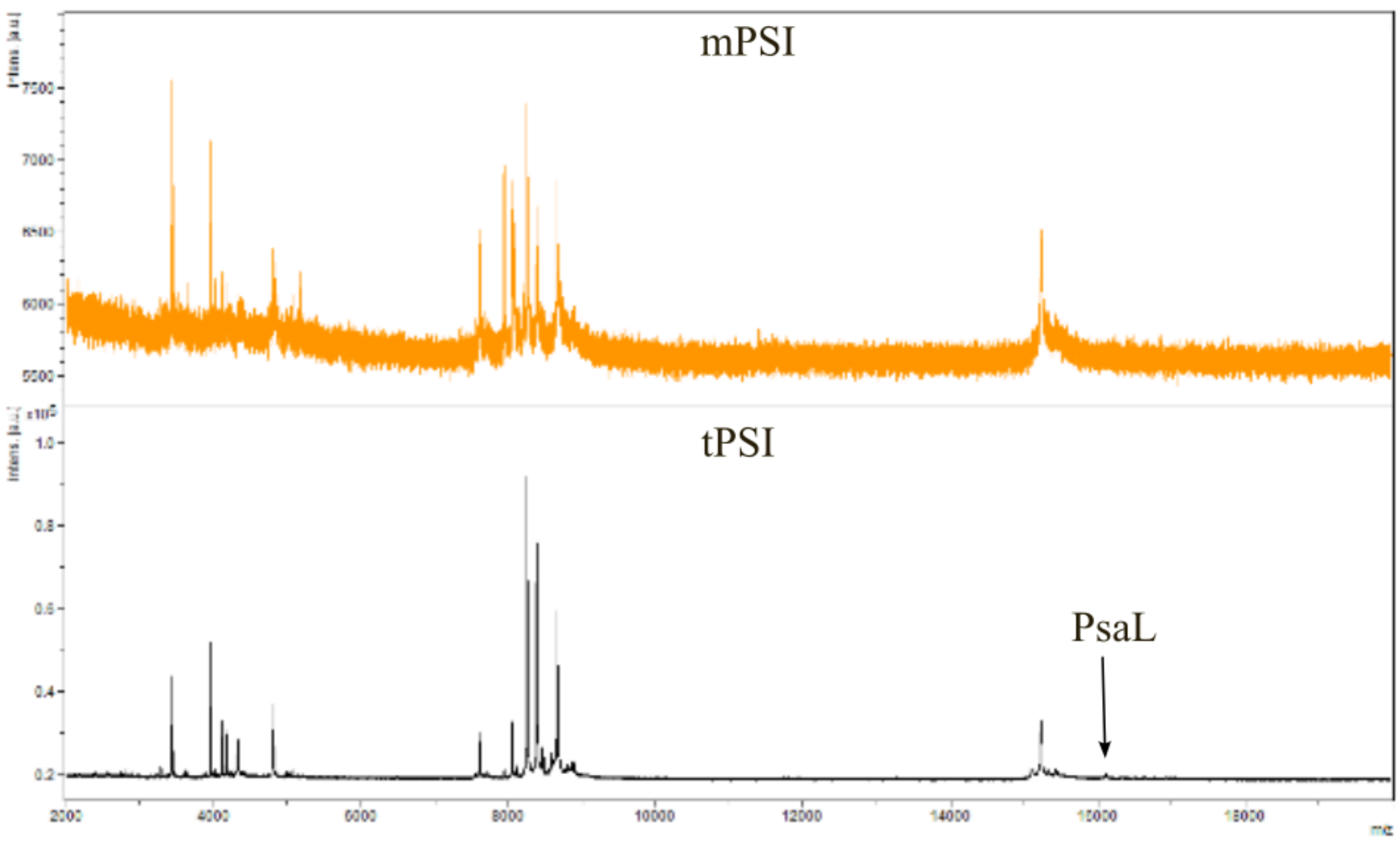

Fig. S4 Mass spectrometry. Exemplary MALDI-ToF spectra of $C_{12}E_8$ mPSI (orange) and tPSI (black) samples show the presence of all subunits below 20 kDa, except for PsaL in the mPSI sample (measured n ≥ 10).

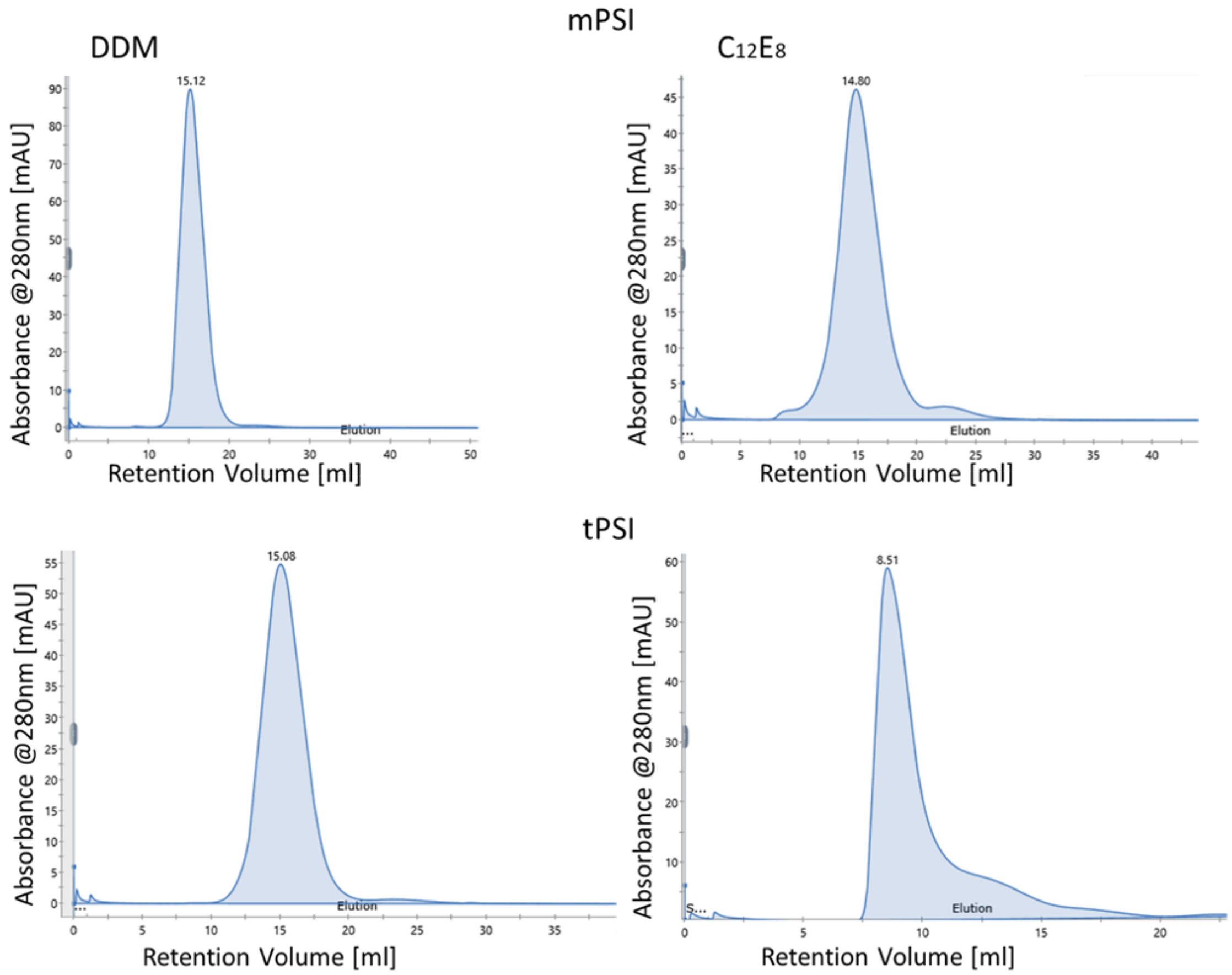

Fig. S5 Exemplary size exclusion chromatograms. mPSI (top row) and tPSI (lower row) purified in either ßDM (left) or $C_{12}E_8$ (right), each n = 3. Due to slightly varying concentrations of the PSI samples, there are differences in the scaling of the axis of each graph. mPSI elutes later ($\bar{x}$ = ßDM 15.1 ml; $C_{12}E_8$ 14.9 ml) than the corresponding tPSI ($\bar{x}$ = ßDM 14.4 ml; $C_{12}E_8$ 8.5 ml) and the $C_{12}E_8$ buffer results in a faster elution time for the protein.

Size exclusion chromatography was performed to ensure sample purity (Fig. S5) after purification. Since the mPSI is smaller in size, it elutes later than tPSI. The concentrations of the applied samples are slightly different, hence the y-axis scaling varies for each graph and the small signals for $C_{12}E_8$ PSI before and after the main peak are in the range of the recorded disturbance from the injection of the sample in the beginning of each elution and can therefore be attributed to the column run.

In DLS experiments, the radius of ßDM mPSI is 4.53 ± 0.07 nm (n ≥ 10) and that of tPSI is 9.47 ± 0.24 nm (n ≥ 3).

Table S1 Sample characterisation. Analysis of tPSI and mPSI ensuring homogeneity, monodispersity (Pd < 15%), size (average retention volume V_R, n = 3; for chromatograms see Fig. S5); hydrodynamic radius R_H, (n ≥ 3, each) and O_2 activity (n ≥ 3, each) in buffer with either 0.02 % ßDM or 0.013 % $C_{12}E_8$.

sample/exp.	V_R [ml]	R_H [nm]	O_2 activity [μmol_{O2} / (mg_{Chl} • h)]
ßDM tPSI	14.4 ± 1	9.47 ± 0.24	-1662[a]
ßDM mPSI	15.1 ± 0.03	4.53 ± 0.07	-1500
$C_{12}E_8$ tPSI	8.5 ± 0.1	9.09 ± 0.30 | 8.9[a]	-2150[a]
$C_{12}E_8$ mPSI	14.9 ± 0.1	4.0[b] ± 1.1	-2120

[a] Values taken from Golub and Boyka, et al. (2025); 0.5 g/l tPSI in buffer with 0.02% ßDM.
[b] Concentration of 0.9 g/l mPSI

Detergent Belt Determination

Small angle neutron scattering (SANS) measurements on our dßDM mPSI are shown in Fig. S6. From these, we calculate a D_{max} of 155± 10 Å as well as a molecular weight (MW) of 300 kDa. Furthermore, we determined the MW of the mPSI together with Cyt c_{HH} at 312 kDa. Further details of the model-independent analysis are provided in Table S2.

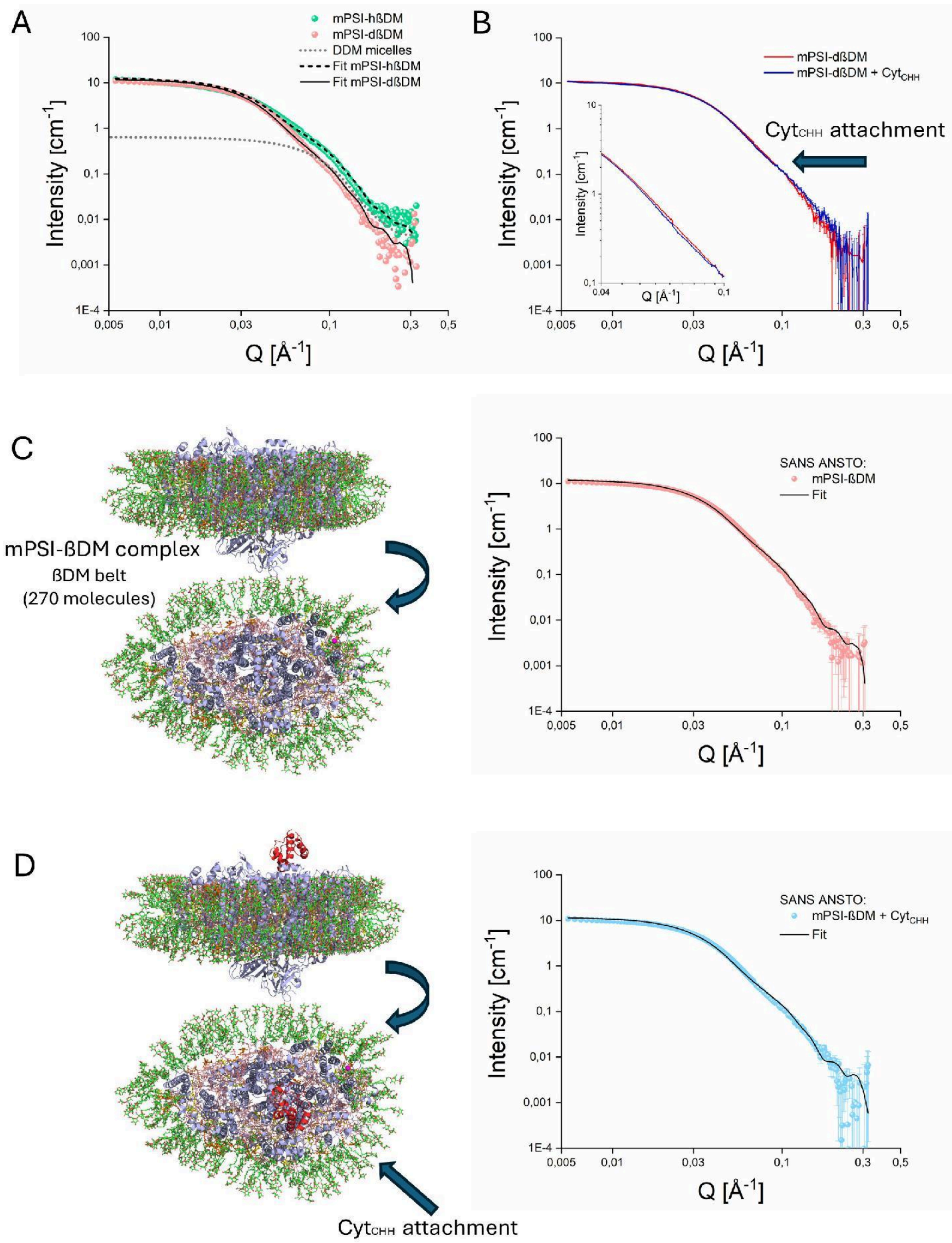

Fig. S6 Small angle neutron scattering.

A: SANS data of mPSI-hßDM (green circles) and of mPSI-dßDM (red circles) complexes. A thin gray dotted line indicates the contribution of free detergent micelles in the case of hßDM before the exchange. The black line is the contribution of the mPSI-dßDM complex. The fit of the mPSI-hßDM SANS data is shown as the dashed black

line describing the sum of scattering contributions from the mPSI-dßDM complex and free ßDM micelles. **B:** SANS data of mPSI-dßDM (red line) and of the mPSI-dßDM + Cyt c_{HH} complex (blue line). The inset shows a magnification of the same data sets in the Q-range, where the data of the mPSI-dßDM + Cyt c_{HH} complex (red line) are shifted towards lower Q, indicating a larger complex (thus indicating binding of Cyt c_{HH}). **C:** *Left*: structural model of the mPSI-dßDM complex based on the cryo-EM structure obtained within this study. *Right*: SANS data of the mPSI-dßDM complex (red circles) and data fit (black line) based on the mPSI-dßDM complex shown in the left panel. **D:** *Left*: structural model of the mPSI-ßDM - Cyt c_{HH} complex based on the cryo-EM structure obtained within this study. *Right*: SANS data of the mPSI-dßDM + Cyt c_{HH} complex (blue circles) and data fit (black line) based on the mPSI-dßDM + Cyt c_{HH} complex shown in the left panel.

We determined the D_{max} and used the program ChimeraX (version 1.7.1) (Goddard et al. 2018; Pettersen et al. 2021; Meng et al. 2023) to calculate the volume of V_{PDC} = 338.4 Å³ and the area of 447.2 Å² of the detergent mPSI complex as described previously (Zampieri et al. 2021). Subtraction of the volume of mPSI without detergent belt at a contour level of 0.0244 ($V_{protein}$ = 131.8 Å³; area=205 Å²) gives a volume of $V_{detergent\ belt}$ = 206.6 Å³ solely of the detergent belt.

For comparison, tPSI shows an R_g of ~ 80 Å in ßDM and 73 Å in $C_{12}E_8$ in SAXS experiments (Golub and Boyka et al. 2025). In addition, the cryo-EM structure of the ßDM mPSI presented here, indicates a maximum interparticle distance (D_{max}) value of 179 Å (D_{min}=120 Å) at a contour level of 0.0121 (Fig. S7, after Zampieri et al., 2021), which correlates well with the D_{max} of 155 ± 10 Å and molecular weight of 300 kDa calculated from our SANS measurements (Fig. S6). These data also fit well to the SANS experiments by Brady et al. (2022), who used styrene maleic acid lipid particles (SMALPs) instead of detergents and determined the $D_{PSI+Lipid}$ of tPSI including its lipid belt to be 334 Å (D_{max} = 354 Å), instead of the estimated 233 Å of the isolated ßDM tPSI (without the detergent belt) and a D_{max} of 291 Å of the ßDM tPSI including the ßDM detergent belt. Therefore, they could derive a ~ 30% larger lipid-PSI complex in SMALPs compared to ßDM tPSI. This aligns well with previous SAXS experiments, in which D_{max} for ßDM tPSI was calculated be to around 270 Å and for $C_{12}E_8$ tPSI at ~ 240 Å (Golub and Boyka et al. 2025).

In addition, we show that the cryo-EM structure of the mPSI-Cyt c_{HH} complex obtained within this study, describes the mPSI SANS data, thus verifying that the high-resolution structure is also valid in solution at room temperature (Fig. S6C).

Table S2. Model independent analysis.

	R_g Guinier [Å]	R_g Gnom [Å]	D_{max} Gnom [Å]	MW [kDa]<br>Size & Shape method
hßDM mPSI	53.29 ± 0.3	51.94 ± 1	179 ± 10	191.130 ± 50
ßDM mPSI	50.37 ± 0.08	51.1 ± 1	155 ± 10	300.272 ± 50
ßDM mPSI with Cyt c_{HH}	50.42 ± 0.19	50.6 ± 1	160 ± 10	311.875 ± 50

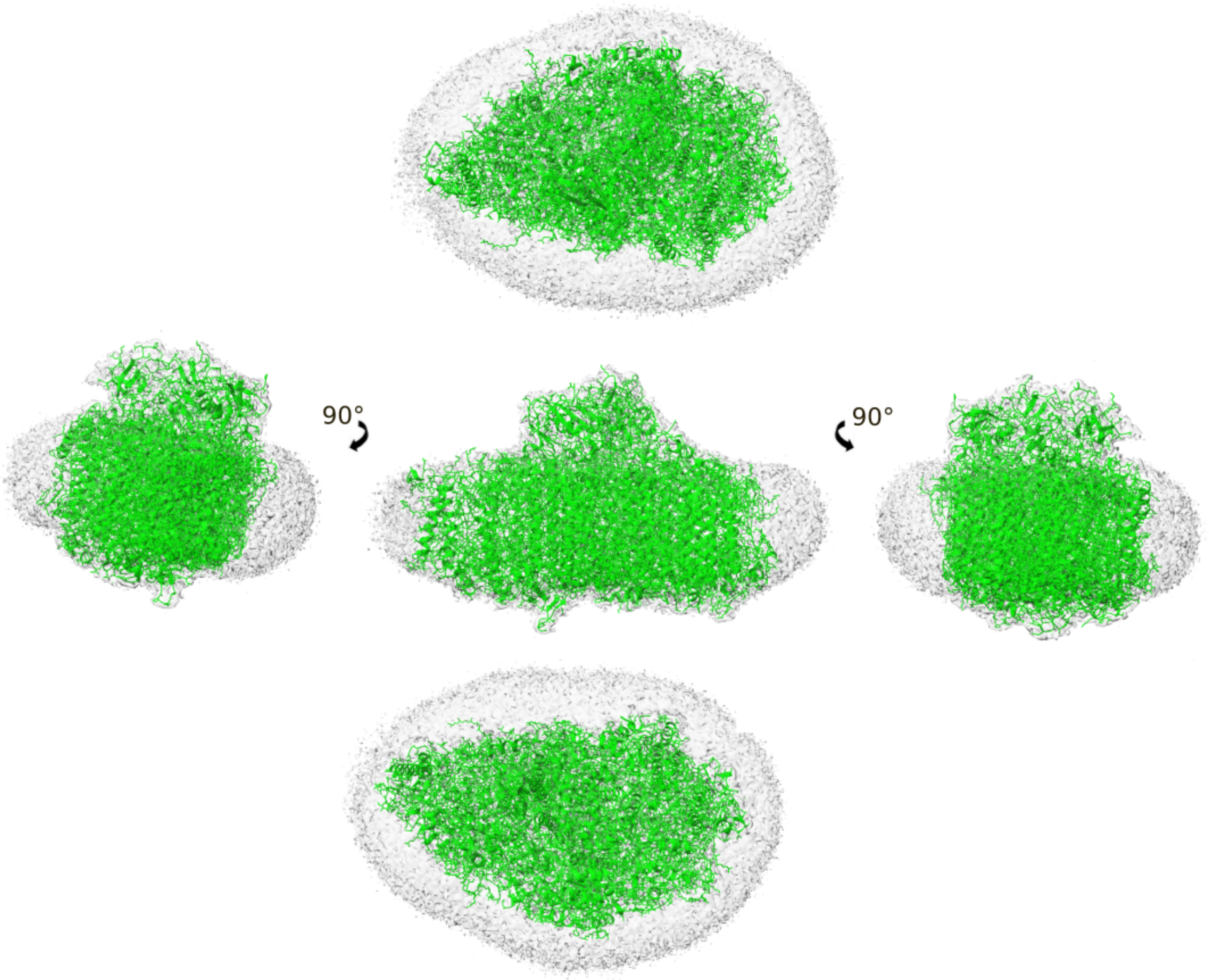

Fig. S7 Cryo-EM volume map. ßDM mPSI (green) with visible detergent belt (grey) and D_{max} = 179 Å at a contour level of 0.0121 (measured after Zampieri et al., 2021).

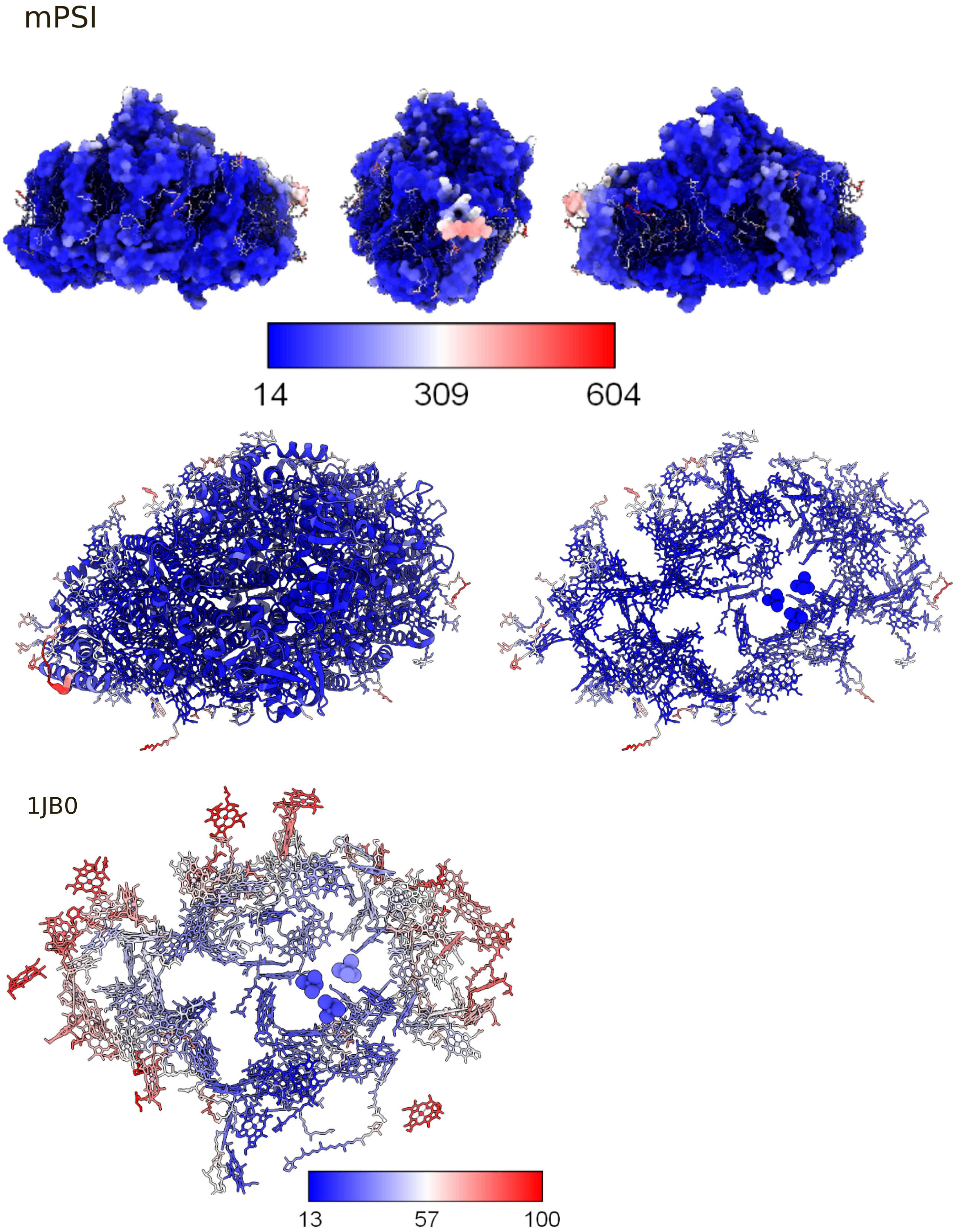

Fig. S8 B-factors of mPSI compared to tPSI. *Top*: mPSI in side view; fixed/clearly resolved regions in blue and more flexible regions in red. PsaK is the most flexible subunit (upper row); the B-factor of our mPSI (middle images: *left* – whole structure; *right* – only cofactors) increases radially from the centre of the monomer to the outer sections. *Bottom*: The monomer in a trimer of 1JB0 shows more rigidity in the central part of the trimer where PsaL is located.

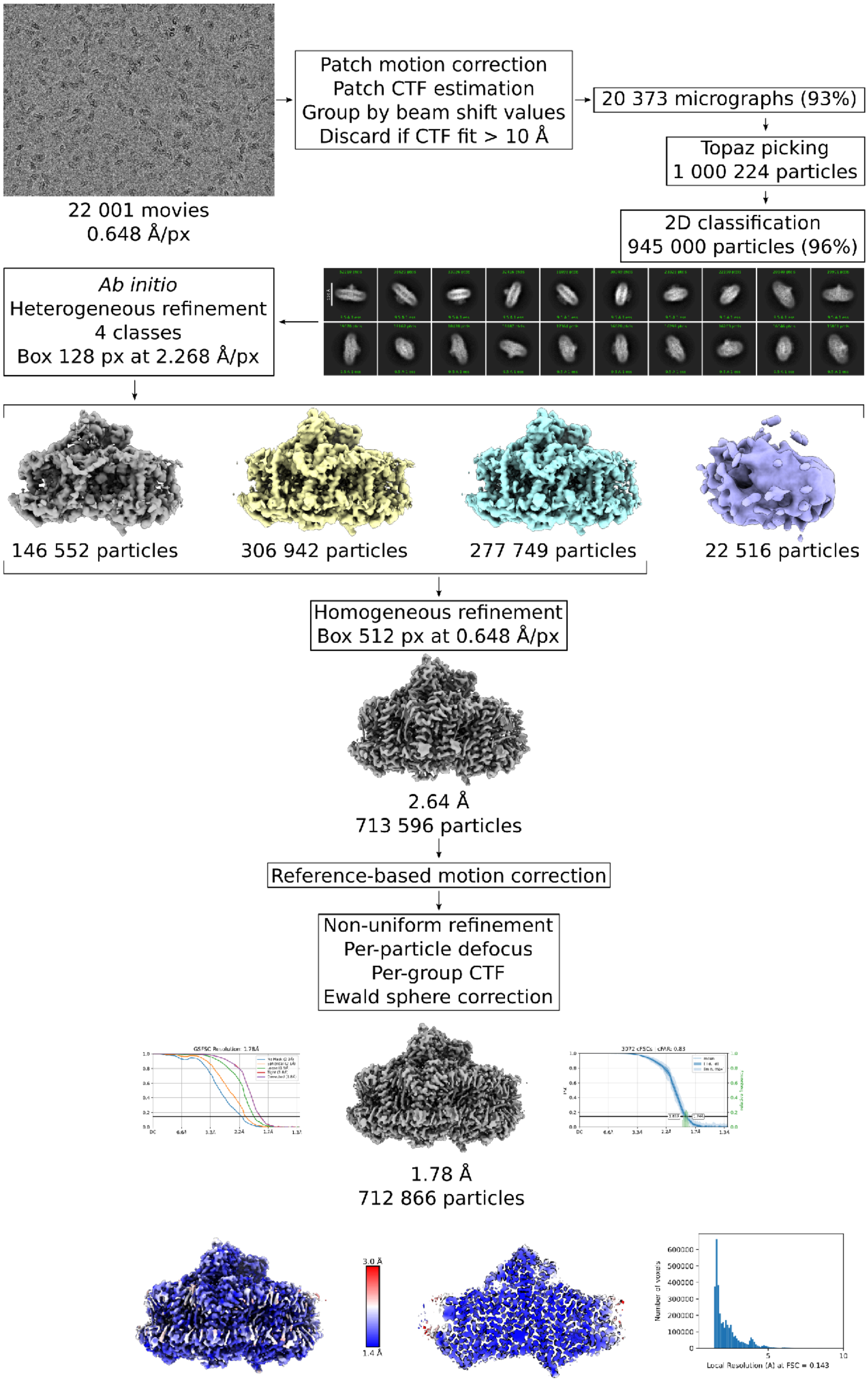

Fig. S9 Flow chart of the cryo-EM image processing.

Table S3. Data collection, image processing and model building and refinement statistics.

EMPIAR	EMPIAR-12814	EMPIAR-13422
EMDB	EMD-53120	EMD-57205
PDB	9QFP	not modelled
**Data collection and image processing**		
Microscope	Krios	Krios
Detector	K3 Bioquantum	Falcon 4i
Energy filter	K3 Bioquantum	Selectris
Energy filter slit width (eV)	20	10
Acceleration voltage (kV)	300	300
Nominal magnification	130000	165000
Pixel size (Å/px)	0.648	0.734
Electron exposure (e^-/Å^2)	40	40
Nominal defocus range (µm)	-0.4 to -1.4	-0.4 to -1.6
Symmetry imposed	C1	C1
Number of exposures collected	22001	13236
Initial particle images (no.)	1000224	184759
Final particle images (no.)	712866	8571
Map resolution (Å)	1.78	2.81
FSC threshold	0.143	0.143
Map resolution range (min/median/max, Å)	1.43 / 1.99 / 22.28	2.35 / 3.64 / 42.93
Map sharpening B factor (Å^2)	-35.8	-37.4
**Atomic model building**		
Initial model used	AlphaFold2 predictions	na
Model-to-map resolution (masked, Å)	1.8	na
Model-to-map FSC threshold	0.5	na
**Model composition**		
Non-hydrogen atoms	25572	na
Protein residues	2134	na
Cofactor or detergent molecules	138	na
Water molecules	1183	na
Mg2+ ions	1	na
**Model validation**		
Bond lengths RMSD (Å)	0.029	na
Bond angles RMSD (°)	3.462	na
MolProbity score	1.6	na
All-atom clashscore	11.7	na
Rotamer outliers (%)	0.81	na
Ramachandran favored (%)	97.92	na
Ramachandran allowed (%)	2.04	na
Ramachandran outliers (%)	0.05	na
Atomic displacement parameters (min/median/max, Å2)	13.93 / 37.50 / 604.06	na
**Model to map fit**		
CC volume	0.9464	na
CC mask	0.9548	na

CC peaks	0.8483	na
Q-scores (min/median/max)	-0.818 / 0.943 / 0.988	na

Table S4. Protein identification from the cryo-EM map.

Uniprot accession code	E-value	Score	Bias	Description
sp|P0A405|PSAA_THEVB	0.0	1962.52	63.22	apoprotein A1 PsaA
sp|P0A407|PSAB_THEVB	0.0	1664.66	63.98	apoprotein A2 PsaB
sp|P0A401|PSAF_THEVB	2.11E-142	467.16	0.89	subunit III PsaF
sp|P0A420|PSAD_THEVB	7.06E-137	449.23	1.37	subunit II PsaD
sp|P0A415|PSAC_THEVB	8.50E-70	231.55	13.34	iron-sulfur center PsaC
sp|P0A423|PSAE_THEVB	8.62E-62	205.57	4.45	subunit IV PsaE
sp|P0A429|PSAJ_THEVB	1.34E-35	120.58	4.14	subunit IX PsaJ
sp|P0A427|PSAI_THEVB	9.95E-30	101.53	6.01	subunit VIII PsaI
sp|P0A425|PSAK_THEVB	3.62E-25	86.73	0.26	PsaK
sp|P0A403|PSAM_THEVB	4.31E-25	86.48	6.91	subunit XII PsaM
tr|Q8DKP6|Q8DKP6_THEVB	3.10E-24	83.70	10.24	PsaX

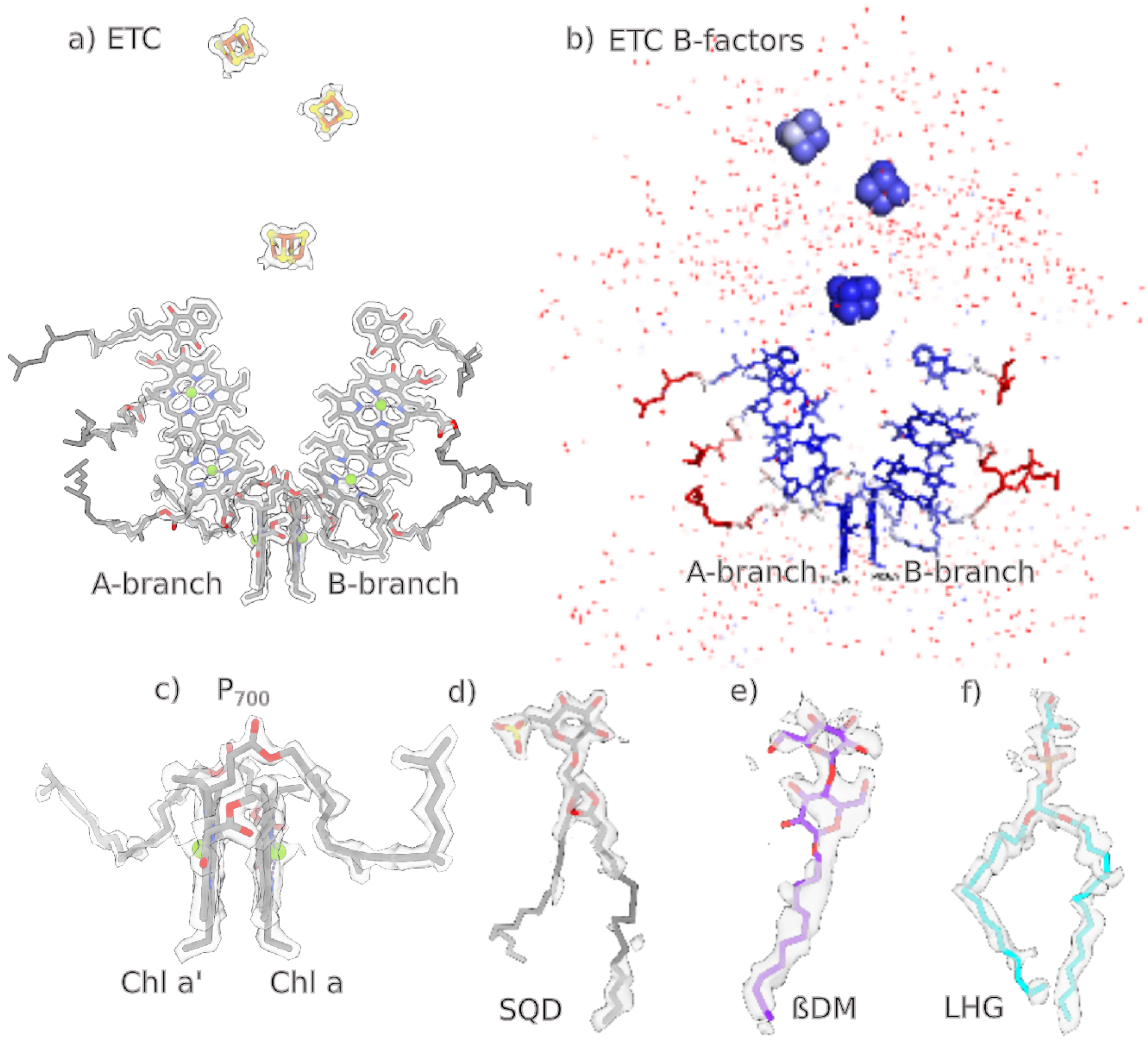

Fig. S10 The ETC and a newly identified SQD. Distances including density (a) and the B-factors (b) in the ETC of ßDM mPSI are similar in both branches, A-branch (left) and B-branch (right), throughout the ETC of our mPSI structure showing less difference between the branches compared to tPSI. Structures of (c) P_{700} Chl *a (right)* and Chl *a*‘ (left), (d) the newly found SQD, (e) ßDM molecule and (f) LHG molecule shown together with density.

Table S5. Coordination and proximity to amino acid residues of ETC cofactors in mPSI. Labelling by cofactors name – branch and residue abbreviation-residue number after pdb.

**Qx-A PQN-837**	**Qx-B PQN-823**
Ser-692 (3.2 Å), Leu-722 (2.9 Å)<br>proximity to Trp-697 (3.4 Å) and Phe-689 (4.8 Å)	Leu-707 (2.9 Å), [only PyMOL: Ser-673 (3.3 Å)]<br>proximity to Trp 678 (3.4 Å)
**Chl *a* (A3) CLA-850**	**Chl *a* (B3) CLA-863**
Tyr-696 (2.6 Å)	Tyr-677 (2.6 Å)
**Chl *a* (B2) CLA-813**	**Chl *a* (A2) CLA-849**
HOH-939 (2.1 Å)	HOH-942 (2.1 Å)
**Chl *a’* (A1) CL0-847**	**Chl *a* (B1) CLA-834**
HOH-1061 (3.2 Å), Tyr-735 (2.6 Å), Thr-743 (2.7 Å) [only PyMOL: Tyr-603 (3.2 Å)]	no H-bonds

Table S6. Comparison of distances in the ETC of different PSI structures. Measurement after Jordan et al. (2001) between chlorophylls from Mg Atom to Mg atom as well as between Mg atom and the centre of the phylloquinone carbonyls (otherwise indicated in parenthesis) and the centre of the iron-sulphur clusters (PyMOL: distances to rings). Abbrev.: *Synechocystis* (*S*), *Zea mays* (*Zm*)

Publication ETC dist. [Å]	mPSI 2026, *Tv* 1.8 Å 9QFP	(Çoruh et al. 2021), *Tv* 3.2 Å 6LU1 cryo-EM	(Jordan et al. 2001), *Tv* 2.5 Å 1JB0 X-ray	(Kölsch et al. 2020), *Tv* 2.85 Å 6TRA cryo-EM	(Keable et al. 2021), *Tv* 2.75 Å 7M75 X-ray	(Li et al. 2022), *Tv* 1.97 Å 7FIX (1st monomer) cryo-EM	(Netzer-El et al. 2018), *S* 4 Å 6HQB X-ray	(Malavath et al. 2018), *S* 2.5 Å 5OY0 (1st monomer) X-ray	(Pan et al. 2018), *Zm* 3.3 Å 5ZJI cryo-EM
P700	5.9	6.0	6.3	6.0	5.7	6.2	5.2	5.6	6.7
**A1-B2**	12.0	12.3	11.7	12.2	11.8	11.8	12.3	11.9	11.4
**B1-A2**	12.2	12.5	12.0	12.2	12.1	12.0	12.3	12.1	11.9
**B2-A3**	8.3	8.5	8.7	8.3	8.4	8.5	8.3	8.5	8.5
**A2-B3**	7.8	8.0	8.2	7.8	9.4	7.9	7.8	7.6	8.0
**A3-Q_A** (to 2nd ring)	9.9 (9.1)	10.3 (9.6)	9.4 (8.6)	10.1 (9.3)	9.6 (9.0)	9.7 (9.0)	10.1 (9.5)	9.8 (9.1)	10.1 (9.4)
**B3-Q_B** (to 2nd ring)	9.7 (8.9)	10.1 (9.4)	9.4 (8.5)	9.9 (9.1)	9.3 (9.0)	9.6 (8.8)	9.6 (9.0)	9.6 (8.9)	9.0 (8.4)
Q_A-F_x (to both/1st ring)	14.2 (13.0/11.9)	14.5 (13.4/12.2)	14.2 (13.0/11.9)	14.3 (13.2/12.0)	14.2 (13.1/12.0))	14.0 (12.8/11.7)	13.9 (12.7/11.5)	14.0 (12.9/11.7)	14.1 (12.9/11.8)
Q_B-F_x (to both/1st ring)	14.2 (13.0/11.9)	14.6 (13.4/12.2)	14.1 (12.9/11.7)	14.0 (12.8/11.7)	14.3 (13.1/12.0)	14.0 (12.8/11.6)	14.2 (13.0/11.8)	14.0 (12.8/11.7)	14.6 (13.4/12.2)
F_x-F_A	15.0	15.8	15.0	15.2	15.4	14.9	14.7	15.0	14.7
F_A-F_B	12.3	12.6	12.3	12.3	12.3	12.1	12.0	11.9	12.5
(F_B-F_x	22.2	23.0	22.0	22.2	22.3	21.8	21.9	21.8	22.0)

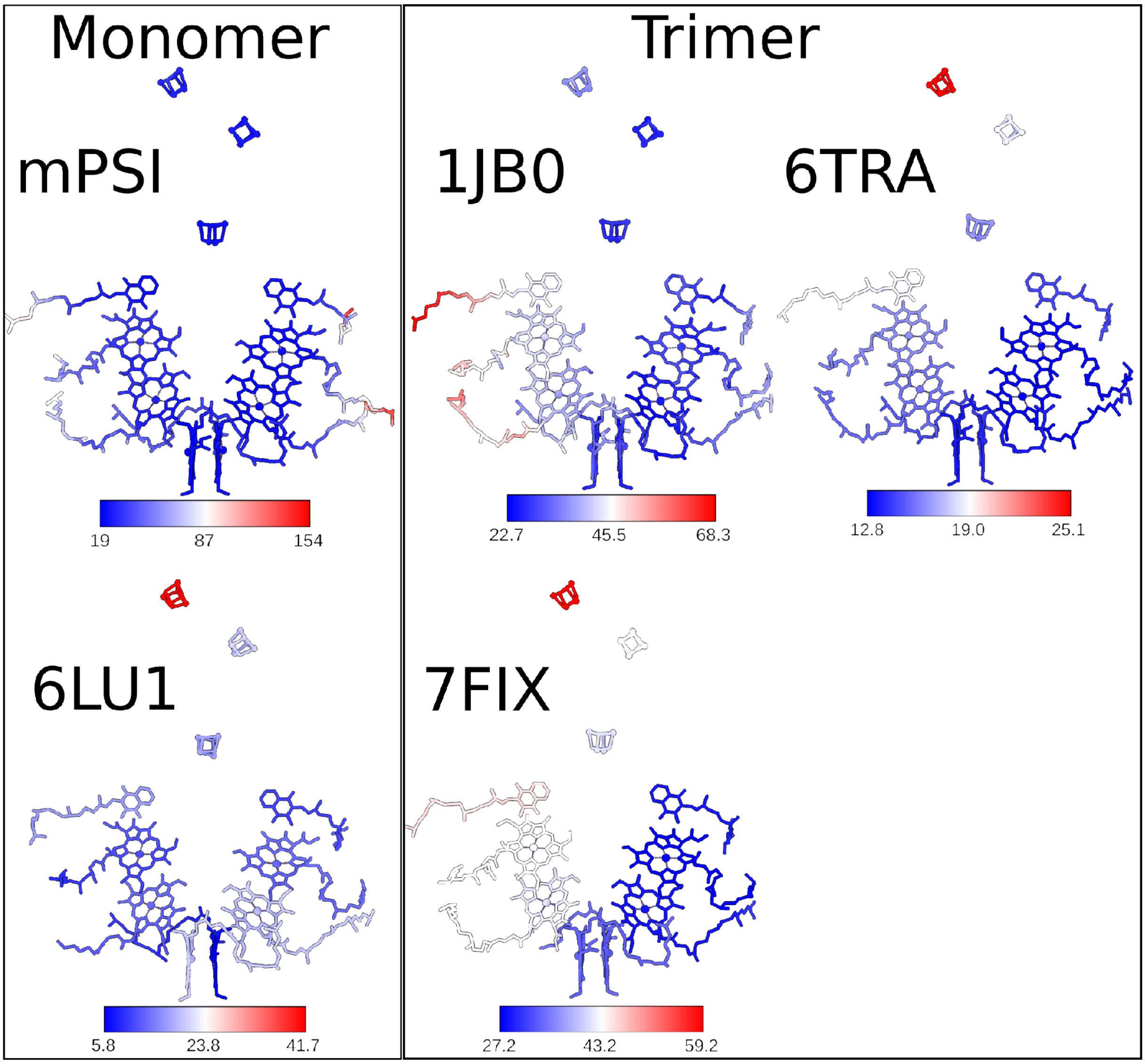

Fig. S11 Comparison of B-factors differences in ETC of PSI. Monomers left (*top*: ours and *bottom*: 6LU1) and trimers right (*top*: 1JB0, 6TRA, *bottom*: 7FIX). Trimeric PSI shows more differences between the ETC branches in rigidity. The A-branch of the trimeric structures are more flexible compared to the B-branch, whereas in monomeric structures both branches are similarly rigid. In a trimer, the A-branch faces radially outwards of the PSI and the B-branch is located closer to the trimerisation region where all three PsaL subunits meet. Furthermore, for all structures except our mPSI, the flexibility of the iron sulphur clusters increases along the direction of the electron transfer from P_{700} to the FeS clusters, with F_B being the most flexible.

In PDB entry 1JB0, the A-branch starting from B2 up to Q_A is more flexible compared to all of the three iron sulphur (FeS) clusters, whereas in the tPSI structure 6TRA, which has a lower resolution, B2 and A3 are similarly rigid to F_A, and Q_A is as flexible as F_A. In all of these trimeric structures and also our mPSI, the P_{700} chlorophylls are both similarly rigid and coordinated by two tyrosines (1JB0 Tyr-603 3.3 Å and Tyr-735 2.8 Å; 6TRA 3.4 Å and 3.0 Å; our mPSI 3.2 Å and 2.6 Å), one threonine (two coordination positions 1JB0 The-743 3 Å and 3.1 Å, 6TRA 3.4 Å and no second; our mPSI 2.7 Å and 3.3 Å), and one water (1JB0 HOH-5011 3.3 Å; our mPSI HOH-1061 3.2 Å). In contrast, the mPSI 6LU1 A1 Chl *a* is far more flexible than B1 and the $C13^2$-$COOCH_3$ group in this monomer structure (Çoruh et al. 2021) was not modelled into the density.

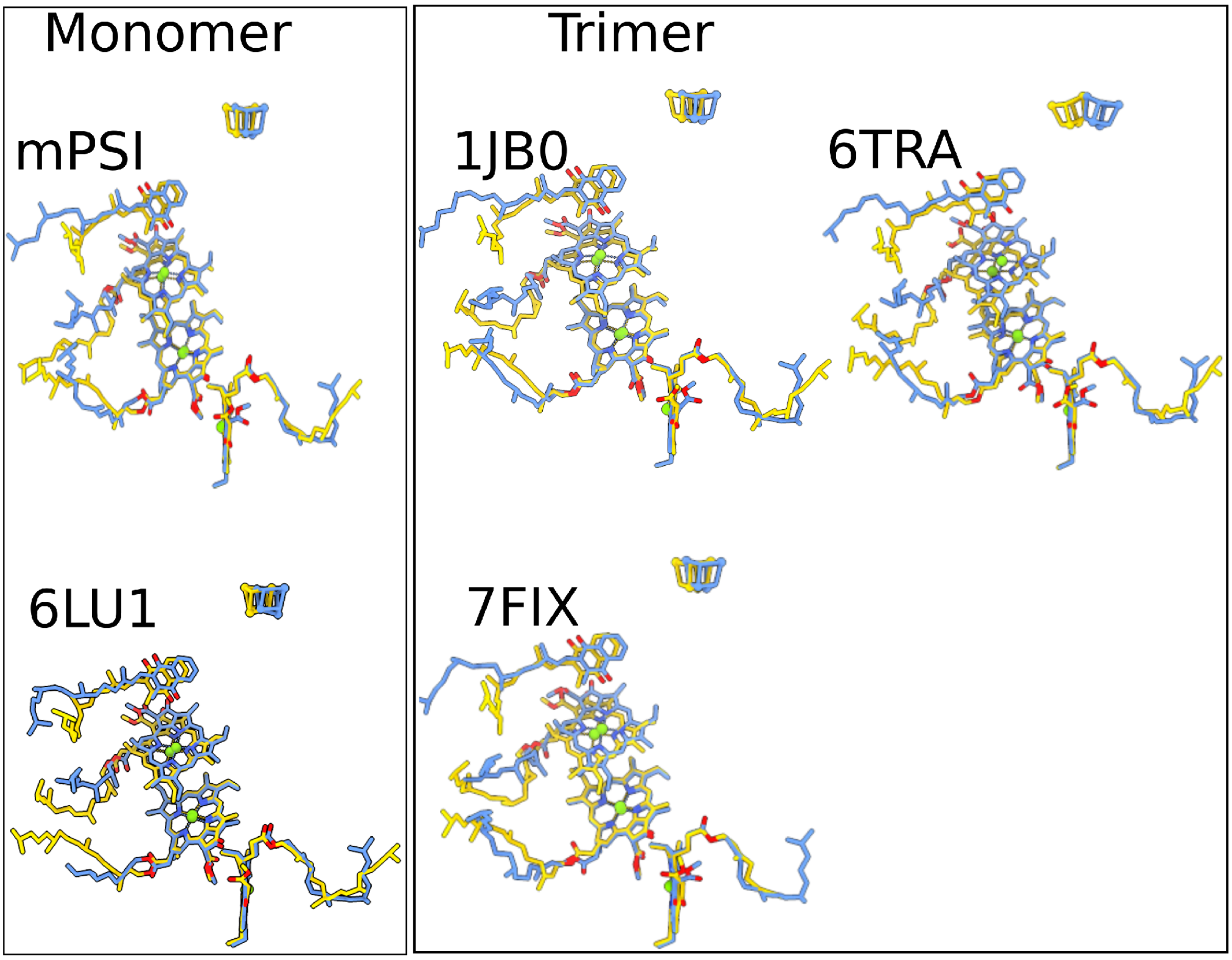

Fig. S12 At the P_{700} superimposed ETC branches (A in blue and B in yellow) of various PSI structures. Monomers left (*top*: ours and *bottom*: 6LU1) and trimers right (*top*: 1JB0, 6TRA, *bottom*: 7FIX). The difference in position of the ETC cofactors at both branches is increasing along the direction of the electron transfer (radially outwards of the trimer) and is higher in trimers (right) compared to monomers (left).

There are no polar coordinating contacts for BCR. Hence, hereafter, we list the BCR pairs closest to each other together with the distance in-between, and Table S7 shows the coordinating molecules of each BCR. BCR-202 and BCR-206 (both PsaF) are at a minimal distance of 3.44 Å from each other. The distance between the following BCR pairs is 3.6 Å: BCR-842 and BCR-838 (PsaA), BCR-862 and BCR-839 (PsaA), PsaK BCR-103 and PsaA BCR-862, BCR-103 and BCR-104 (PsaJ). In addition, BCR-839 and BCR-841 (both PsaA) as well as BCR-802 and BCR-803 (both PsaB) are 3.7 Å apart, PsaA BCR-840 and PsaF BCR-202 3.8 Å, PsaJ BCR-103 and PsaA BCR-840 3.9 Å, BCR-822 and BCR-825 (both PsaB) as well as BCR-803 and BCR-824 (both PsaB) 4 Å.

In most cases, the conjugated π-systems of Chl molecules are at a distance of ~ 5 Å from the BCR molecules; only one distance is 4 Å.

Table S7. ß-carotene contacts identified via ChimeraX. Listed are only molecules at a distance < 3.5 Å; # contacts only indicated if more than one.

Subunit: BCR	Coordinating cofactor (# contacts, closest distance to BCR)	Coordinating amino acids
A: 838	CLA-806 (2, 3.4 Å), CLA-811 (2, 3.4 Å), CLA-818 (3.3 Å), CLA-833 (5, 3.2 Å), PsaK: CLA-104 (3.4 Å)	
839	CLA-809 (3.36 Å), CLA-817 (3.48 Å), CLA-822 (3.47 Å)	Ala-211 (3.43 Å)
840	CLA-808 (3.2 Å)	Trp-744 (2, 3.3 Å), Phe-681 (3.45 Å)
841	CLA-809 (3.46 Å), CLA-810 (3.47 Å), CLA-815 (3.3 Å)	
842	CLA-805 (6, 3.2 Å), CLA-832 (3.5 Å), CLA-849 (3.45 Å), PsaB: CLA-841 (3.3 Å)	
862	CLA-859 (3.46 Å), CLA-861 (3.47 Å)	Phe-267 (3.46 Å)
B: 802<br>803<br>822<br>824	CLA-833 (3.4 Å)<br>CLA-811 (2, 3.4 Å), CLA-831 (3.35 Å)<br>CLA-808 (3, 3.4 Å), CLA-810 (3.45 Å)<br>CLA-814 (2, 3.4 Å), CLA-817 (3.4 Å), CLA-818 (3.4 Å), CLA-831 (3.44 Å)	
825	CLA-849 (4, 3 Å), CLA-846 (3.47 Å)	Trp-340 (3.46 Å)
826	CLA-841 (3.3 Å), PsaA: CLA-832 (3.5 Å), CLA 849 (3.45 Å)	
F: 202	CLA-204 (3.5 Å), BCR-206 (3.4 Å), PsaA: CLA-850 (3.45 Å), CLA-858 (3.4 Å), PsaB: CLA-805 (2, 3.4 Å), CLA-842 (3.38 Å)	Ile-96 (3.48 Å)
203<br>206	PsaX: CLA-102 (4, 3.3 Å)<br>BCR-202 (3.44 Å), PsaJ: CLA-101 (4, 3.37 Å)	Val-76 (2, 3 Å)<br>Arg-60 (3.34 Å), Pro-89 (3.47 Å), PsaB: Phe-432 (3.44 Å), PsaJ: His-39 (2, 3.2 Å)
K: 103	CLA-101 (7, 3.3 Å)	Thr-63 (3.1 Å), His-67 (2, 3.45 Å)
J: 103<br>104	PsaA: CLA-856 (7, 3 Å), PsaB: CLA-842 (3.49 Å)<br>CLA-857 (3.3 Å), HOH-1243 (2, 3.3 Å), PsaJ: CLA-102 (3.4 Å)	 Tyr-7 (3.3 Å)
M: 101		Leu-16 (3.4 Å), Ala-19 (3.5 Å), Ala-22 (3.45 Å)

Table S8. Coordination of the lipids PG (LHG) and MGDG (LMG) found in our mPSI.
contacts only mentioned if > 1.

Subunit: lipid molecule	Coordinating cofactor (# contacts, closest distance)	Coordinating amino acids
PsaA: LHG-843		Thr-337 (2, 2.6 Å), His-332 (2, 2.85 Å), Phe-336 (3 Å)
LHG-864	LMT-803 (2, 2.8 Å)	
LHG-852	HOH-919 (2, 2.5 Å), HOH-937 (2.5 Å), HOH-981 (2.6 Å), HOH-1081 (2.8 Å), HOH-1155 (3 Å), HOH-1195 (3.2 Å), PsaE: HOH-135 (3.3 Å)	Arg-575 (2, 3 Å), Trp-592 (3 Å), Ser-723 (2.6 Å)
PsaI: LHG-101	HOH-203 (3.2 Å)	
PsaX: LHG-104	PsaF: HOH-337 (3.3 Å)	Tyr-13 (2, 2.7 Å), PsaA: Lys-710 (3.2 Å)
PsaB: LMG-804	HOH-935 (2.8 Å), HOH-	Ala-563 (3.36 Å), Trp-580 (2.9 Å)

Table S9. Detergent molecules (ßDM = LMT) of our mPSI together with their coordinating molecules (separated into cofactors and amino acid residues). The most prevalent listed hereafter by decreasing order: Chls, waters and other detergents or lipids.

Subunit: det. (LMT) molecule	Coordinating cofactor (# contacts, closest distance to LMT)	Coordinating amino acids
PsaA: 801<br>802<br>803<br>804<br>834<br>835<br>836<br>845	HOH-980 (1, 2.6 Å),<br>LMT-835 (2, 2.8 Å)<br>LHG-864 (2, 2.8 Å)<br>PsaK: LMT-102 (2, 3.0 Å)<br>CLA-831 (2, 2.6 Å)<br>LMT-802 (2, 2.8 Å), CLA-822 (2, 2.9 Å)<br>No coo, proximity (3.8 Å) to CLA-807<br>CLA-844 (2, 2.7 Å) & HOH-1207 (1, 3.5 Å)	Gln-472 (2, 2.7 Å), Asp-473 (2.8 Å)<br><br><br><br><br><br><br>Trp-118 (3 Å)
PsaF: 205	PsaJ: HOH-208 (1, 3.3 Å)	PsaJ: Lys-2 (3.5 Å), Ser-9 (2.9 Å), Thr-6 (3.1 Å), PsaF: Val-126 (2.9 Å), Ile-123&124 (2.8 Å&3.2 Å)
827<br>851<br><br>852	CLA-818 (2, 2.9 Å)<br>PsaB: HOH-946 (1, 2.5 Å), PsaM: HOH-203 (1, 3 Å)<br>No coo, proximity to PsaB: CLA-819 (3.2 Å) [, SQD-101 3.9 Å]	 PsaB: Gln-158 (2, 2.5 Å), PsaJ: Glu-28 (3.4 Å), PsaM: Lys-31 (3.1 Å)<br>No coo, proximity to PsaB: Ile-488 (3.7 Å), [PsaX: Leu-29, 4.2 Å]
PsaB: 801	HOH-920 (1, 2.4 Å), HOH-1177 (1, 2.9 Å), PsaA: CLA-827 (1, 3.3 Å)	
PsaK: 102	Psa-A: 804 (2, 3.0 Å)	
105		PsaK: Tyr-32 (2, 2.6 Å), Ala-33 (2.8 Å), Gln-35 (2.7 Å)
PsaX: 103	No coo, proximity to PsaB: HOH-969 (3.7 Å), CLA-806 (3.6 Å)	No coo, proximity to Phe-35 (3.3 Å), PsaF: Arg-79 (3.5 Å)

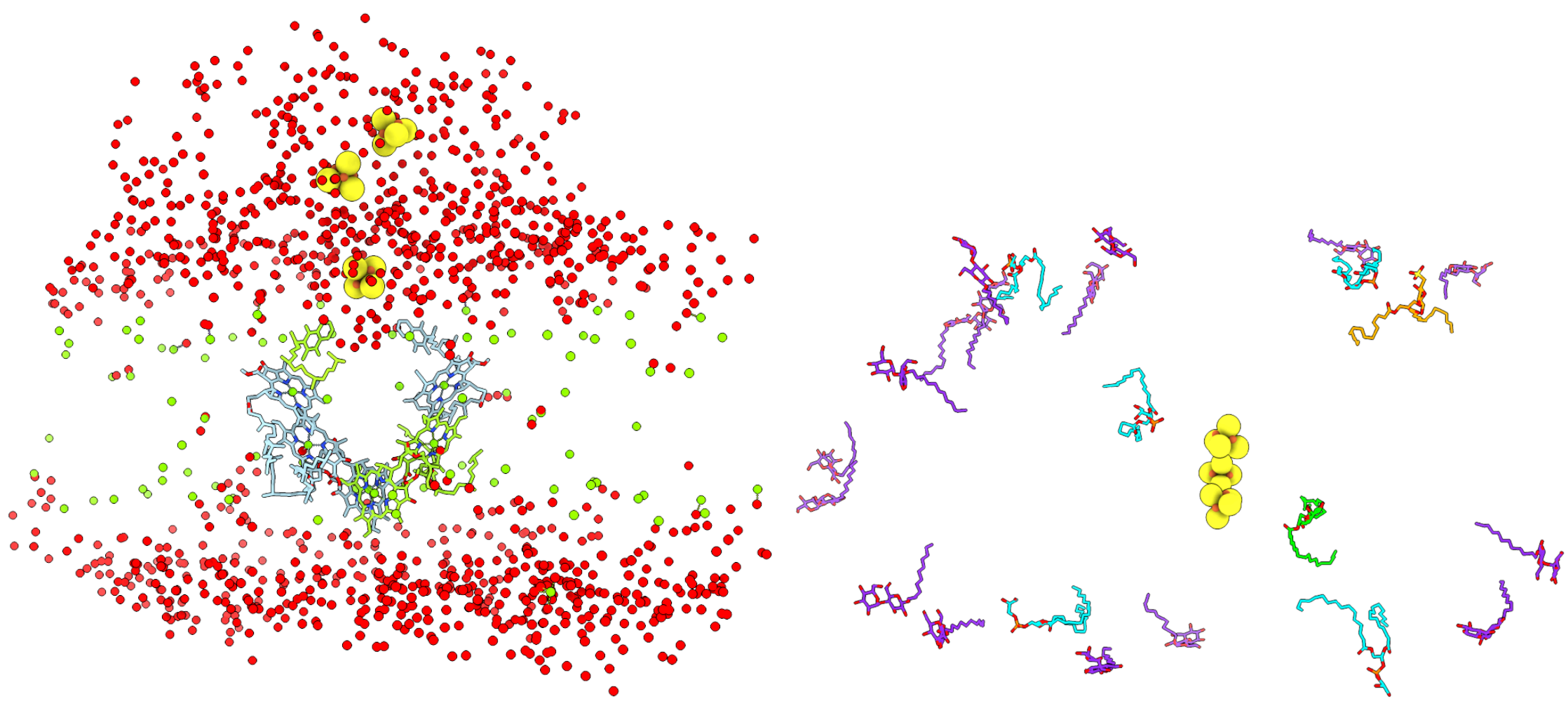

Fig. S13 *Left*: Localisation of Mg-atoms from Chl (green dots) and 35 - 50 water molecules (red dots) in estimated membrane plane with 15 coordinations of water molecules; membrane view (PsaM in front) includes the ETC (PsaA molecules in blue and PsaB in green, FeS clusters in yellow/orange). *Right*: lipid and detergent molecules in our mPSI structure (view from cytoplasm; ßDM in violet, LHG in cyan, SQD in orange, LMG in green).

Water molecules

With 1183 water molecules, our mPSI structure exhibits the highest number of waters detected in PSI up to date and shows their arrangement outside of the membrane plane either luminal (~ 490) or cytoplasmatic (~ 615-635). The trimer structure 5OY0 (Malavath et el., 2018) identified 889 water molecules in total, which would be roughly 296 waters per monomer (this corresponds to about one quarter of the water molecules we found in our structure). Moreover, we do not observe a clustering of waters near the FeS. Instead, there is a cluster of 11 waters in-between the phylloquinones and F_x similar to what is seen in the structure 5OY0.

Table S10. Comparison of water molecules in different PSI structures.

**Max. distance [Å] to our mPSI (1.8 Å, 1183 H_2O)**	**1JB0 (2.5 Å, 201 H_2O)**	**6TRA (2.85 Å, 217 H_2O/m)**	**6LU1 (3.2 Å, 5 H_2O)**	**7FIX (1.97 Å, 116 H_2O/m)**	**5OY0 (2.5 Å, 889 H_2O/t)**
0.1	5	0	0	0	2
0.5	123	40	0	46	111
1	154	114	2	85	**N.d.**
Difference in resolution to 9QFP	(0.7 Å) 147	(1.05 Å) 118	(1.4 Å) 5	(0.17 Å) 1	(0.7 Å) 170
% of total waters align	73	54	100	< 1	57

From the five waters in 1JB0 deviating only 0.1 Å from our mPSI structure and hence are at a similar position in both structures, three are close to PQN A-837 namely A-1107 (3.8 Å), A-1038 (3.5 Å) and A-1075 (3.9 Å) [1JB0: PQN A-2001 and A-HOH 5007 (3.7 Å), A-HOH 5015 (3.5 Å) and A-5043 (3.9 Å)]. The other two are A-1169 (1JB0 A-5014) and B-1023 (1JB0 B-5040).

When comparing the only other structure of *Tv* mPSI to our results, only two waters at a distance of 1 Å are at the same position B-1159 (6LU1: B-902) and C-1142 (6LU1: D-201) and five at 1.4 Å, including: A-1038 (6LU1: A-901), A-1125 (6LU1: B-901) and C-1159 (6LU1: C-201).

Only one water molecule is in the distance of the structure resolution differences (0.17 Å) with 7FIX namely B-935 (7FIX: B-909) and for the *Synechocystis* structure 5OY0 (0.7 Å) we find 170 water molecules at a similar position to our mPSI. Furthermore, two water positions are within 0.1 Å of 5OY0 and our structure, namely A-976 (5OY0: A-8081) and B-1130 (5OY0: B-8082).

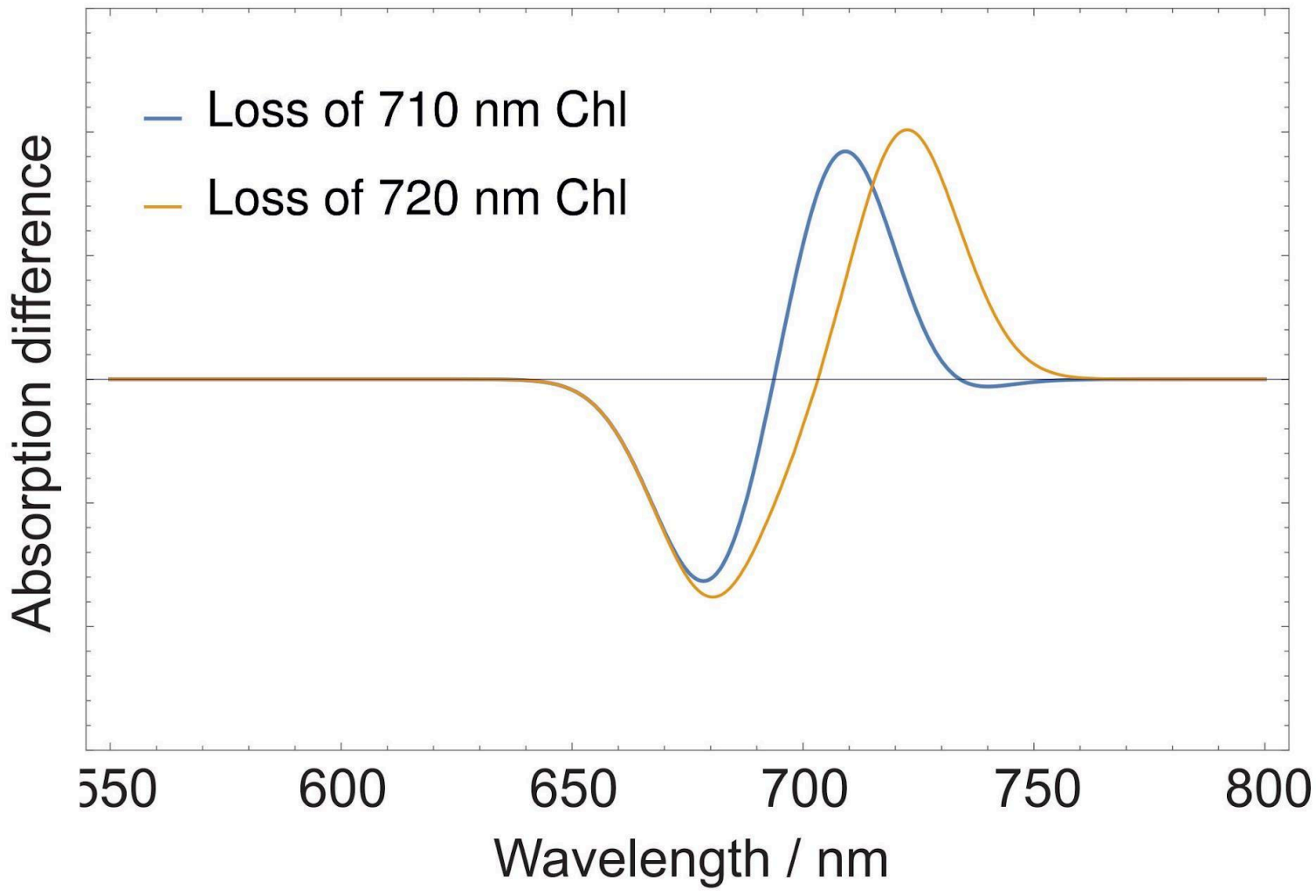

Fig. S14 Simplistic modelling of tPSI-minus-mPSI absorption difference spectra. Modelled absorption differences as a result of losing besides four "normal" Chls also a "red" Chl, which absorbs either at 710 nm (similar to 708 nm; blue) or 720 nm (similar to 719 nm; orange), upon monomerisation (absorption spectra normalised at 620 nm). Our measured tPSI-minus-mPSI difference spectra look more similar to the loss of a 708 nm Chl.

Table S11. Red Chls after Schlodder et al. (2007). Residue numbers according to 1JB0 (Jordan et al. 2001) and to our mPSI in parenthesis with chain indication if it is differing.

Chain	Chl Tetramer	Chl Trimer	Chl Dimer	Chl Monomer
PsaA	1131 (832), 1132 (848),	1102 (854), 1103 (814), 1104 (819); 1119 (824), 1120 (859), 1121 (807)	1138 (853), 1139 (F-201); 1126 (820), 1127 (815); 1237 (851)	
PsaB	1206 (840), 1207 (missing)	1231 (838), 1232 (846), 1233 (819)	1238 (839); 1224 (829), 1225 (814)	1211 (833)

Table S12: Comparison of P_{700} absorption maxima, fluorescence emission peak maxima at 77 K. mPSI and tPSI purified in buffer containing either 0.02% ßDM or 0.013% $C_{12}E_8$, each n ≥ 3. Fluorescence emission maxima (2) of (averaged) samples prepared either in $C_{12}E_8$ or ßDM after excitation at 440 nm and adjusted to c_1 = 2.5 µg / ml or c_2 = 120 µg / ml Chl in buffer containing the noted detergent concentration.

	**P_{700} absorption peak [nm]**	**0.02% ßDM or 0.013% $C_{12}E_8$**	**0.5% ßDM**	**0.5% $C_{12}E_8$**	**1% ßDM**	**1% $C_{12}E_8$**
**ßDM tPSI c_1**	680	674, 728	675, 728	673, 727	673, 728	672, 727
**c_2**		731.9 ± 0.6	732 ± 1	732.4 ± 1.2	731.6 ± 1.3	731.2 ± 1.3
**ßDM mPSI c_1**	678.5	674, 726	673, 724	673, 726	674, 725	674, 725
**c_2**		728.1 ± 0.8	727.6 ± 0.9	730.2 ± 1.2	728.1 ± 1.4	728.8 ± 1.4
**$C_{12}E_8$ tPSI**	679	672, 728	673, 728	673, 728	672, 729	673, 730
**$C_{12}E_8$ mPSI**	675.5	673, 724	673, 724	673, 727	673, 725	673, 727

Table S13: Thermal stability. CD transition temperatures ($T_{transition}$) of ßDM- and $C_{12}E_8$-purified tPSI and mPSI with varying detergent content in the buffer indicate that a lower thermal stability correlates with higher $C_{12}E_8$ concentrations in the buffer (n ≥ 3, each).

	**CD $T_{transition}$ [°C]**			
% detergent in buffer	**0.02% ßDM**	**0.013% $C_{12}E_8$**	**1% ßDM**	**1% $C_{12}E_8$**
**ßDM tPSI**	88.7 ± 1.4	-	93.1 ± 5.5	56.7 ± 0.5
**ßDM mPSI**	89.1 ± 8.2	-	85.4 ± 6.8	55.0 ± 2.7
**$C_{12}E_8$ tPSI**	-	64.0 ± 1.2	99.6 ± 5.4	59.1 ± 0.7
**$C_{12}E_8$ mPSI**	-	56.5 ± 1.5	67.6 ± 2.2	54.9 ± 1.9

Table S14. Comparison of different monomeric (m) and trimeric (t) PSI structures from cyanobacteria and higher plants to our mPSI structure (9QFP). Abbrev.: chlorophyll *a* (Chl *a*, CLA), phylloquinone (PQN), carotenoids (Car), ß-carotene (BCR), phosphatidyl glycerol (PG, LHG), monogalactosyl diacylglycerol (MGDG, LMG), sulfoquinovosyl diacylglycerol (SQDG, SQD), digalactosyl diacylglycerol (DGDG, DGD), Not modelled (N.m.).

Publi-cation	(Jordan et al. 2001)	(Kölsch et al. 2020)	(Keable et al. 2021)	(Malavath et al. 2018)	(Toporik et al. 2020)	(Gisriel et al. 2019)	(Li et al. 2022)	(Ben-Shem et al. 2003)	(Çoruh et al. 2021)	(Pan et al. 2018)	Our model
pdb	1JB0	6TRA	7M75	5OY0	6UZV	6PGK	7FIX	1QZV	6LU1	5ZJI	9QFP
organism	*T. vestitus*	*T. vestitus*	*T. vestitus*	*Synechocystis sp.* PCC6803	*Synechocystis sp.* PCC 6803 *red_A mutant*	*T. vestitus*	*T. vestitus*	*Pisum sativum var. alaska*	*T. vestitus*	*Zea mays subsp. mays*	*T. vestitus*
oligomeric state	t	t	t	t	t	t	t	m	m	m	m
resolution	2.5 Å	2.85 Å	2.75 Å	2.5 Å	3.1 Å	2.9 Å	1.97 Å	4.44 Å	3.2 Å	3.3 Å	1.8 Å
method	x-ray diff.	cryo-EM	RT XFEL	x-ray diff.	cryo-EM	RT XFEL	cryo-EM	x-ray diffraction	cryo-EM	cryo-EM	cryo-EM
subunits	12	12	12	11/33 (-PsaD)	12	12	12	12	10 (-PsaX, -PsaK)	12 (+PsaN, PsaO)	11 (-PsaL)
Cofactors:	127/m			127/m		381 (same as in 5OY0)		Only Chl a, PQN modelled	112/m		
Chl *a*	96/m			285/t =95/m (85 in PsaA&B)			285/t =95/m, 3x Cla'/t	167	82 (-14)	143 (+2 in PsaN & +2 PsaO; +8 PsaX; +8 PsaY; +8 PsaZ) (7740/8709)	91 (-5) 3 of PsaL, 1 PsaJ, 1 PsaI(7fix)= PsaB (1jB0)=PsaL (6tra) 1jb0/6tra: 1 PsaM

												(not in 7fix)
Pqn	2/m	2/m	2/m	2/m	2/m	2/m	6/t=2/m	2/m	2/m	2/m	2/m
Fe_4S_4	3/m	3/m	3/m	3/m	3/m	3/m	9/t=3/m	3/m	3/m	3/m	3/m
Car	22/m 865atoms			72			72	N.m.	26 (+8 new, -4 lost) (932 atoms)	26 BCR, 5(+6) LUT, 4(+3) XAT	19 (-4 at PsaL, +1 BCR1004 in PsaK and BCR4009 from 1JB0 fully modelled) 760 atoms
lipids: LHG LMG SQD DGD	4/m (3 LHG (5001, 3-4), 1LMG(5002))	6/m (5 LHG 101-2, 851-3, 1 LMG 850)	9/m (4 LHG (853-4, 101, 3049), 4 LMG (104, 852, 855, 3048), 1 DGD (206)	51/t 16LMG, 27 LHG/t, 7SQD/t, 1DGD/t	8/m (4 LHG (101, 801-2, 852), 4 LMG (803, 852-3, L-1506),	4/m 3 LHG (), 1 LMG (846)	51/t 1DGD/m, 3LHG/m,	N.m.	3 (2LHG 850-1, 1LMG 843), lost lipid IV	12(+3)/m (5(+3) LHG, 5LMG, 2DGD)	5xLHG 1xLMG 1xSQD
detergent LMT (ßDM)	-	-	1 ßDM/t LMT	4 ßDM/t LMT	2 ßDM / m (F-202, I-4101)	-		N.m.	-	2 LMU/m	16xßDM (LMT)
ions	(putative) 1 L-Ca^{2+}/m	2(L+B)Ca^{2+} /m	2(L+B) Ca^{2+}/m	6(L+B)Ca^{2+} /t, 1Mg^{2+}/t, 3 Cl^-/t	3 L-Ca^{2+}/t	3L-Ca^{2+}/t	3 L-Ca^{2+}/t	N.m.	-	-	1 Mg^{2+}/m
waters	201/m	217/m	119/m	889/t ≈296/m	-	71/m	348/t =116/m	N.m.	5/m	-	1183/m

References

Ben-Shem A; Frolow F; Nelson N (2003): Crystal structure of plant photosystem I. In: Nature 426 (6967):630–635. https://doi.org/10.1038/nature02200.

Brady NG; Qian S; Nguyen J; O'Neill HM; Bruce BD. (2022): Small angle neutron scattering and lipidomic analysis of a native, trimeric PSI-SMALP from a thermophilic cyanobacteria. In: Biochimica et Biophysica Acta (BBA) - Bioenergetics 1863 (7):148596. https://doi.org/10.1016/j.bbabio.2022.148596

Çoruh O; Frank A; Tanaka H; Kawamoto A; El-Mohsnawy E; Kato T; Namba K; Gerle C; Nowaczyk MM; Kurisu G (2021): Cryo-EM structure of a functional monomeric Photosystem I from Thermosynechococcus elongatus reveals red chlorophyll cluster. In: Commun Biol 4 (1):304. https://doi.org/10.1038/s42003-021-01808-9.

Gisriel C; Coe J; Letrun R; Yefanov OM; Luna-Chavez C; Stander NE; Lisova S; Mariani V; Kuhn M; Aplin S; Grant TD; Dörner K; Sato T; Echelmeier A; Cruz Villarreal J; Hunter M S; Wiedorn MO; Knoska J; Mazalova V; Roy-Chowdhury S; Yang J; Jones A; Bean R; Bielecki J; Kim Y; Mills G; Weinhausen B; Meza JD; Al-Qudami N; Bajt S; Brehm G; Botha S; Boukhelef D; Brockhauser S; Bruce BD; Coleman MA; Danilevski C; Discianno E; Dobson Z; Fangohr H; Martin-Garcia JM; Gevorkov Y; Hauf S; Hosseinizadeh A; Januschek F; Ketawala GK; Kupitz C; Maia L; Manetti M; Messerschmidt M; Michelat T; Mondal J; Ourmazd A; Previtali G; Sarrou I; Schön S; Schwander P; Shelby ML; Silenzi A; Sztuk-Dambietz J; Szuba J; Turcato M; White TA; Wrona K; Xu C; Abdellatif MH; Zook JD; Spence JCH; Chapman HN; Barty A; Kirian RA; Frank M; Ros A; Schmidt M; Fromme R; Mancuso AP; Fromme P; Zatsepin NA (2019): Membrane protein megahertz crystallography at the European XFEL. In: Nat Commun 10 (1):5021. https://doi.org/10.1038/s41467-019-12955-3.

Goddard TD; Huang CC; Meng EC; Pettersen EF; Couch GS; Morris JH; Ferrin TE (2018): UCSF ChimeraX: Meeting modern challenges in visualization and analysis. In: Protein Science 27 (1):14–25. https://doi.org/10.1002/pro.3235.

Golub M and Boyka J; Gätcke J; Hart O; Haupt S; Wieland DCF; Blanchet CE; Zouni A; Pieper J (2025): Detergent Choice Shapes the Solution Structures of Photosystems I and II: Implications for Crystallization and High-Resolution Studies. In: J. Phys. Chem. B 129 (33):8392–8405. https://doi.org/10.1021/acs.jpcb.5c00767.

Jordan P; Fromme P; Witt HT; Klukas O; Saenger W; Krauss N (2001): Three-dimensional structure of cyanobacterial photosystem I at 2.5 A resolution. In: Nature 411 (6840):909–917. https://doi.org/10.1038/35082000.

Keable SM; Kölsch A; Simon PS; Dasgupta M; Chatterjee R; Subramanian SK; Hussein R; Ibrahim M; Kim IS; Bogacz I; Makita H; Pham CC; Fuller FD; Gul S; Paley D; Lassalle L; Sutherlin KD; Bhowmick A; Moriarty NW; Young ID; Blaschke JP; Lichtenberg C; Chernev P; Cheah MH; Park S; Park G; Kim J; Lee SJ; Park J; Tono K; Owada S; Hunter MS; Batyuk A; Oggenfuss R; Sander M; Zerdane S; Ozerov D; Nass K; Lemke H; Mankowsky R; Brewster AS; Messinger J; Sauter NK; Yachandra VK; Yano J; Zouni A; Kern J (2021): Room temperature XFEL crystallography reveals asymmetry in the vicinity of the two phylloquinones in photosystem I. In: Sci Rep 11 (1):21787. https://doi.org/10.1038/s41598-021-00236-3.

Kölsch A; Radon C; Golub M; Baumert A; Bürger J; Mielke T; Lisdat F; Feoktystov A; Pieper J; Zouni A; Wendler P (2020): Current limits of structural biology: The transient interaction between cytochrome

c6 and photosystem I. In: Current Research in Structural Biology 2:171–179. https://doi.org/10.1016/j.crstbi.2020.08.003.

Li J; Hamaoka N; Makino F; Kawamoto A; Lin Y; Rögner M; Nowaczyk MM; Lee YH; Namba K; Gerle C; Kurisu G (2022): Structure of cyanobacterial photosystem I complexed with ferredoxin at 1.97 Å resolution. In: Commun Biol 5 (1):951. https://doi.org/10.1038/s42003-022-03926-4.

Malavath T; Caspy I; Netzer-El SY; Klaiman D; Nelson N (2018): Structure and function of wild-type and subunit-depleted photosystem I in Synechocystis. In: Biochimica et Biophysica Acta (BBA) - Bioenergetics 1859 (9):645–654. https://doi.org/10.1016/j.bbabio.2018.02.002.

Meng EC; Goddard TD; Pettersen EF; Couch GS; Pearson ZJ; Morris JH; Ferrin TE (2023): UCSF ChimeraX: Tools for structure building and analysis. In: Protein Science 32 (11), e4792. https://doi.org/10.1002/pro.4792.

Netzer-El SY; Caspy I; Nelson N (2018): Crystal Structure of Photosystem I Monomer From Synechocystis PCC 6803. In: Front Plant Sci 9:1865. https://doi.org/10.3389/fpls.2018.01865.

Pan X; Ma J; Su X; Cao P; Chang W; Liu Z; Zhang X; Li M (2018): Structure of the maize photosystem I supercomplex with light-harvesting complexes I and II. In: Science 360:1109-1113. https://www.science.org/doi/10.1126/science.aat1156.

Pettersen EF; Goddard TD; Huang CC; Meng EC; Couch GS; Croll TI; Morris JH; Ferrin TE (2021): UCSF ChimeraX: Structure visualization for researchers, educators, and developers. In: Protein science : a publication of the Protein Society 30 (1):70–82. https://doi.org/10.1002/pro.3943.

Schlodder E; Shubin VV; El-Mohsnawy E; Roegner M; Karapetyan NV (2007): Steady-state and transient polarized absorption spectroscopy of photosystem I complexes from the cyanobacteria Arthrospira platensis and Thermosynechococcus elongatus. In: Biochimica et biophysica acta 1767 (6):732–741. https://doi.org/10.1016/j.bbabio.2007.01.013.

Toporik H; Khmelnitskiy A; Dobson Z; Riddle R; Williams D; Lin S; Jankowiak R; Mazor Y (2020): The structure of a red-shifted photosystem I reveals a red site in the core antenna. In: Nat Commun 11 (1):5279. https://doi.org/10.1038/s41467-020-18884-w.

Zampieri V; Gobet A; Robert X; Falson P; Chaptal V (2021): CryoEM reconstructions of membrane proteins solved in several amphipathic solvents, nanodisc, amphipol and detergents, yield amphipathic belts of similar sizes corresponding to a common ordered solvent layer. In: Biochimica et biophysica acta. Biomembranes 1863 (11):183693. https://doi.org/10.1016/j.bbamem.2021.183693.
